# Protein Stability and Dynamics Modulation: The Case of Human Frataxin

**DOI:** 10.1371/journal.pone.0045743

**Published:** 2012-09-25

**Authors:** Ernesto A. Roman, Santiago E. Faraj, Mariana Gallo, Andres G. Salvay, Diego U. Ferreiro, Javier Santos

**Affiliations:** 1 Instituto de Química y Físico-Química Biológicas, Universidad de Buenos Aires, Buenos Aires, Argentina; 2 Fundación Instituto Leloir and IIBBA-CONICET, Buenos Aires, Argentina; 3 Instituto de Física de Líquidos y Sistemas Biológicos, Facultad de Ciencias Exactas, Universidad Nacional de La Plata, La Plata, Argentina; 4 Departamento de Ciencia y Tecnología, Universidad Nacional Quilmes, Bernal, Provincia de Buenos Aires, Argentina; 5 Protein Physiology Laboratory, Departamento de Química Biológica-CONICET, Facultad de Ciencias Exactas y Naturales, Universidad de Buenos Aires, Buenos Aires, Argentina; National Institute for Medical Research, Medical Research Council, United Kingdom

## Abstract

Frataxin (FXN) is an α/β protein that plays an essential role in iron homeostasis. Apparently, the function of human FXN (hFXN) depends on the cooperative formation of crucial interactions between helix α1, helix α2, and the C-terminal region (CTR) of the protein. In this work we quantitatively explore these relationships using a purified recombinant fragment hFXN90–195. This variant shows the hydrodynamic behavior expected for a monomeric globular domain. Circular dichroism, fluorescence, and NMR spectroscopies show that hFXN90–195 presents native-like secondary and tertiary structure. However, chemical and temperature induced denaturation show that CTR truncation significantly destabilizes the overall hFXN fold. Accordingly, limited proteolysis experiments suggest that the native-state dynamics of hFXN90–195 and hFXN90–210 are indeed different, being the former form much more sensitive to the protease at specific sites. The overall folding dynamics of hFXN fold was further explored with structure-based protein folding simulations. These suggest that the native ensemble of hFXN can be decomposed in at least two substates, one with consolidation of the CTR and the other without consolidation of the CTR. Explicit-solvent all atom simulations identify some of the proteolytic target sites as flexible regions of the protein. We propose that the local unfolding of CTR may be a critical step for the global unfolding of hFXN, and that modulation of the CTR interactions may strongly affect hFXN physiological function.

## Introduction

**Figure 1:**
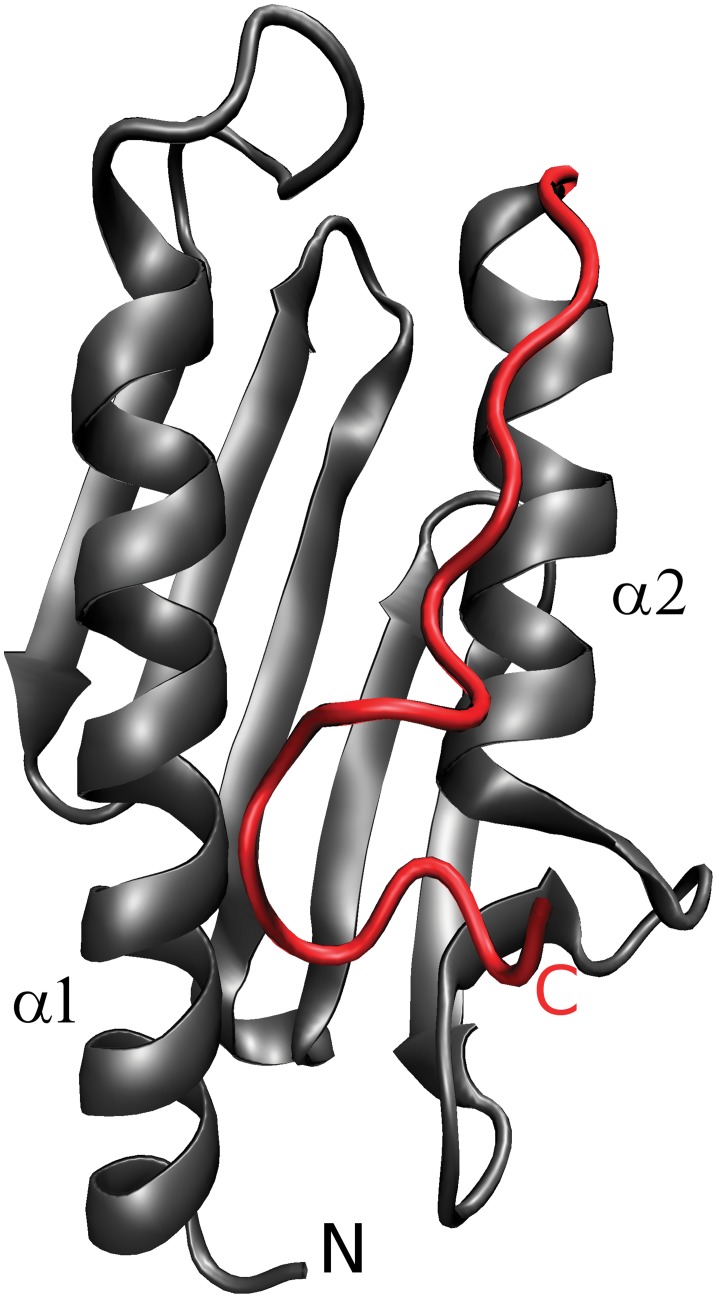
Ribbon representation of the hFXN structure. CTR shown in red interacts with helix-α1 and helix-α2. For a detailed description of these interactions see Table S2.

Friedreich Ataxia (FRDA) is a hereditary disease that affects children and adolescents characterized by progressive neurological impairment and cardiomyopathy [1], [2], [3], [4]. FRDA is highly associated with a deficiency in the expression of the frataxin protein (FXN) [5], [6], [7], [8]. This protein is expressed in the cytoplasm and imported into the mitochondria [9], [10], [11], [12], where it plays an essential role in iron homeostasis. It is believed that FXN acts as an iron chaperone delivering Fe (II) to enzyme partners during heme and Fe-S cluster biosynthesis [13], [14], [15], [16], [17], [18], [19], [20], [21]. hFXN is synthesized as a precursor polypeptide of 210 amino acids. This precursor contains an N-terminal transit sequence that directs its transport into the mitochondria matrix where it is cleaved by the mitochondrial processing peptidase to the mature form FXN81–210. [11] This processing involves an intermediate form (hFXN42–210) [22]. In addition, hFXN 56–210 and hFXN78–210 can be generated *in vivo* when the normal maturation process of FXN is impaired, although the physiological relevance of these forms is unclear [11]. It has been suggested by bioinformatics [23] and shown by nuclear magnetic resonance (NMR) [24], [23], that the N-terminal of hFXN, including the first nine residues of the mature form, has an intrinsically unfolded character. In addition, flexibility of this region is also suggested by the absence of a detectable electron density map for residues 84–89 in the X-ray structure (PDB = 1EKG) [25]. The protein used in this work corresponds to the evolutionarily conserved C-terminal domain of hFXN (amino acids 90–210, the wild-type variant hFXN90–210) [26]. The average native structure of the human protein (hFXN) has already been resolved by NMR and crystallography (Figure 1) [25], [27], [28]. FXN is an α/β protein with a five-stranded, antiparallel β sheet that forms a flat “platform”, and two parallel α helices that are tightly packed against it forming an αβ sandwich.

In about 5% of FRDA point mutations in hFXN gene were reported. Few structural and functional details are known for ∼15 different missense mutations identified in FRDA patients. However, some of them have been studied in depth showing major differences in their thermodynamic stability (e.g. stability of wild-type > W155R > I154F > D122Y > G130V), propensity to aggregate (mutants I154F and W155R precipitate upon iron binding), and function (mutants D122Y and G130V have a lower binding Fe^2+^ stoichiometry) [26], [29]. Interestingly, despite the apparent differences in stabilities, NMR studies have shown that these mutants retain a compact core and native-like dynamics [26]. In addition, three FRDA missense mutations N146K, Q148R, and R165C were also thoroughly studied showing that, in these cases, hFXN is functionally compromised in binding and activation of the SDUF complex (consisting of proteins NFS1, ISD11, ISCU2, and FXN) for Fe−S cluster biosynthesis [28].

Adinolfi et al. proposed that variations on the C-terminal region (CTR) correlate with the conformational stability of different homologues (e.g., yeast, *E. coli*, human) [30], [31]. In addition, they observed that the deletion of the CTR of *E. coli* FXN yields an overall less stable protein (T_m_ is 14°C lower than the T_m_ of the full-length protein). They also remodeled the C-terminal of the yeast FXN by extending it on the basis of the interactions identified in *E. coli* and hFXN CTRs. This yielded a yeast FXN variant considerably more stable as judged by thermal denaturation. Inspired in this work, we decided to further investigate the role of human CTR in stability and dynamics of hFXN.

In the hFXN, CTR region encompasses ∼15 (residues 196 to 210) that, in the native form, pack against helices α1 and α2 occluding the apolar side chains of L198, L200, L203 and Y205 (Figure 1). However, little is known about the contribution of CTR to the conformational stability of the hFXN. Since some FXN homologs do not display the CTR, we speculate that this segment may contribute to the physiological role by altering the detailed protein folding dynamics.

Here, we hypothesize that the stability of hFXN depends on the formation of a cooperative tertiary contacts network based on the modulation of the interaction between α-helix 1 and α-helix 2 by the CTR, making a sort of conformational “lock”. In this work we explored experimentally and computationally the effect of the deletion of the residues 196–210 of the hFXN.

## Results

### Protein Expression

#### hFXN90–195 was produced from inclusion bodies, refolded and further purified

When the truncated variant hFXN90–195 is expressed in *E coli,* most of the protein remains in inclusion bodies (IBs), the insoluble fraction. IBs were prepared and then resuspended in 3.0 M urea. In this condition, hFXN90–195 is efficiently solubilized. Next, hFXN90–195 was refolded by dialysis against buffer 20 mM Tris-HCl, 100 mM NaCl, pH 7.0, and further purified in native conditions as described in Materials and Methods. This protocol results in >95% purity as ascertained by sodium dodecyl sulfate-polyacrylamide gel electrophoresis (SDS-PAGE), reversed-phase high-performance liquid chromatography (RP-HPLC) and electrospray ionization mass spectrometry (ESI-MS). In these conditions, the protein remains highly soluble after refolding and purification and it can be concentrated up to 35 mg/mL without noticeable aggregation, suggesting that this fragment may acquire a compact conformation.

#### hFXN90–195 is monomeric in solution

In order to study the oligomeric state of hFXN variants, analytical ultracentrifugation (AUC) and dynamic light scattering measurements were performed as a function of protein concentration (Figure S1 and Tables 1 and S1). The Stokes radius (R_S_) obtained by AUC for hFXN90–210 was of 1.9±0.1 nm, a value compatible with the R_S_ predicted from hFXN structure (1.86 nm). In addition, the R_s_ demonstrated to be nearly invariant in the range of 0.25 to 1.00 mg/mL. On the other hand, the R_s_ value obtained for the hFXN90–195 was 1.8±0.1 nm, which is also consistent with the value expected for a protein of this size and with a globular shape in solution (1.78 nm). Moreover, both variants showed a frictional ratio of f/fmin = 1.25 (Figure S1). Altogether, these results indicate that both hFXN90–195 and hFXN90–210 behave as compact monomers with a globular hydrodynamic behavior. DLS analysis of variant hFXN90–210 revealed homogeneous samples. The autocorrelation functions are well described by a single-exponential decay that corresponds to a primarily monomodal distribution without polydispersity (%Pd = 14.4), and both intensity and mass corresponding to the peak were 100%. On the other hand, DLS analysis of variant hFXN90–195 revealed some degree of polydispersity (%Pd = 28.7) that can be explained by the presence of a small fraction of molecules (∼10%) with an expanded or unfolded conformation (∼2.5–3.0 nm) in solution (Figure S2). In addition, the peak corresponded to 97% of the total intensity and 100% of the total mass.

**Table 1:** Hydrodynamic Radii of hFXN Variants hFXN90–210 and hFXN90–195.

Hydrodynamic Radii		
Method	hFXN90–210 (nm)	hFXN90–195 (nm)
Dynamic Light Scattering (experimental)	1.89±0.05	1.82±0.03
Analytical Ultracentrifugation (experimental)	1.90±0.1	1.80±0.1
Prediction using the Molecular Mass a	1.86	1.78
Hydropro (theoretical)^b^	1.98	–

a Assuming a globular shape, the R_S_ of the native state of a given protein may be calculated using the equation log (R_S_) = -(0.254±0.002)+(0.369±0.001)×log (MW), where MW is the molecular weight expressed in Da and the hydrodynamic radius is in Å according to reference [60].

b Calculated using Hydropro Software according to reference [67].

### Spectroscopic Characterization of Fragment hFXN90–195

#### hFXN90–195 is well-folded and exhibits native-like tertiary structure

**Figure 2:**
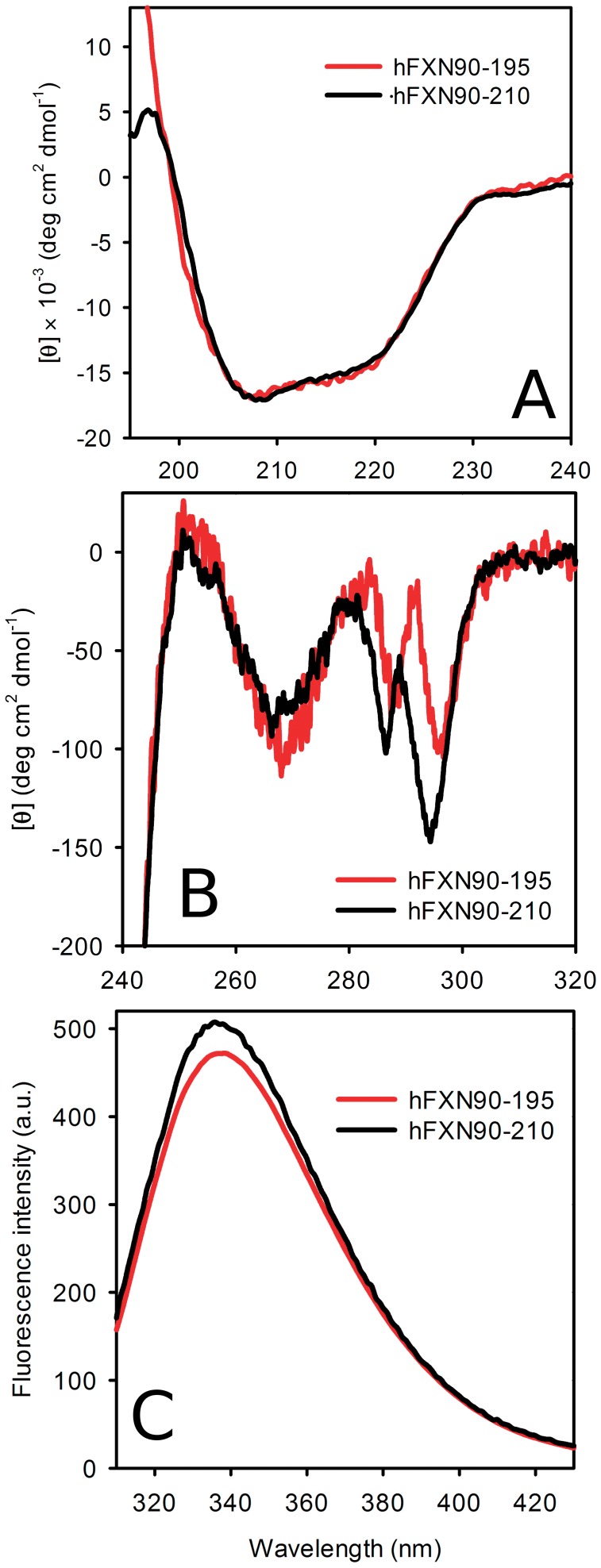
Spectroscopic characterization of the truncated variant. (A) Far-UV CD, (B) near-UV CD spectra and (C) tryptophan fluorescence emission spectra. The measurements were carried out at 20°C in buffer 20 mM Tris·HCl, 100 mM NaCl, 1 mM EDTA, pH 7.0.

To determine the secondary structure content of hFXN90–195, far-UV CD spectra of both variants were acquired and compared (Figure 2A). As judged by the shape and the intensities of the signals, the secondary structure of the C-terminal truncated variant is native-like. In addition, hFXN90–195 near-UV CD spectrum is compatible with the existence of a substantial chirality as a consequence of asymmetry in the vicinity of aromatic residues, suggesting a native-like tertiary structure (Figure 2B). Furthermore, tryptophan fluorescence spectra of both variants have similar features: maximal wavelength emission compatible with the emissions from an apolar environment (Figure 2C), signature of a structurally conserved and dehydrated protein core.

**Figure 3:**
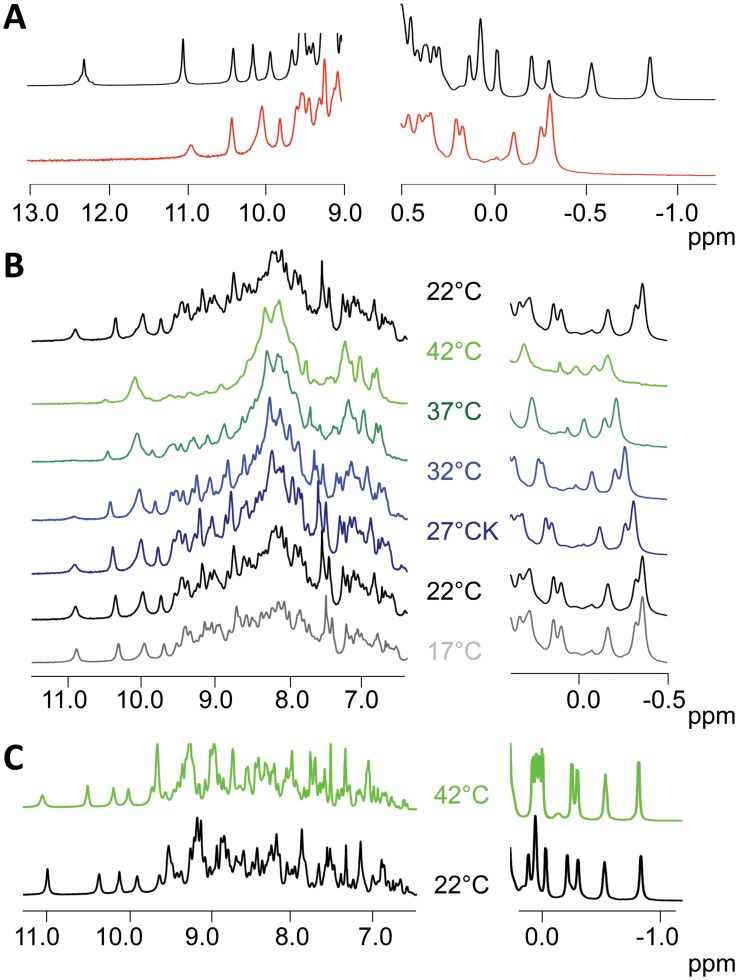
NMR characterization of the truncated variant. (**A**) Selected regions of the ^1^H NMR spectra of hFXN90–210 (red) and hFXN90–195 (black). NMR spectra were performed at 22°C and the rest of the spectroscopic data was acquired at 20°C. All protein solutions were prepared in buffer containing 20 mM Tris·HCl, 100 mM NaCl, 1 mM EDTA, pH 7.0. ^1^H NMR spectra of hFXN90–195 at different temperatures from 17 to 42°C are shown (**B**). The protein loses chemical shift dispersion and exhibits a broadening of signals as the temperature increases. The process is reversible, recovering the 22°C spectrum after having heated the sample to 42°C. (**C**) ^1^H NMR spectra of hFXN90–210 at 22 and 42°C. Both spectra are characteristic of a well-folded globular protein.

The NMR proton spectrum of the full length hFXN shows a high-quality peak dispersion (Figure 3A), evidence of the globular and stable native conformation of the wild-type protein [27]. On the other hand, the proton spectrum of hFXN90–195 shows high field methyl signals at negative chemical shifts (−0.20, −0.35 and −0.40 ppm) and a good signal dispersion in the amide region of the spectrum, exhibiting several signals at chemical shifts larger than 9.0 ppm (Figure 3A). This indicates that the fragment behaves as a well-folded protein. However, there are substantial differences between the spectra of both proteins. The full-length hFXN exhibits an NMR spectrum of higher quality, displaying, for example, methyl signals at −0.5 and −1.0 ppm and a low field signal at 12.3 ppm. These signals are not present in the shorter construct. This suggests that hFXN90–195 would explore less compact conformations when compared to the full-length hFXN in the same timescales. In addition, the presence of overlapped peaks with a lower spreading of chemical shifts in the ^1^H-^15^N HSQC spectra of variant hFXN90–195 is compatible with the coexistence of a small fraction of protein in an unfolded conformation (data not shown).

A substantial reduction in the intensities of the methyl and low field signals in the proton NMR spectrum of hFXN90–195 was observed as the temperature increased from 17 to 42°C. Simultaneously, signals between 7.8 and 8.4 ppm became more intense and an inhomogeneous line width is noticeable in the spectra at higher temperatures (Figure 3B). These changes suggest partial unfolding of the protein when the temperature increases. The process was fully reversible (Figure 3B, upper spectrum), and is further confirmed by thermal unfolding followed by CD (see below). In contrast, hFXN90–210 at 42°C exhibited only minor changes in the NMR proton spectrum in relation to 22°C (Figure 3C), consistent with the higher thermal stability of the full-length protein.

### Reversible Unfolding and Protein Stability

#### The truncation of the CTR destabilizes the hFXN

**Figure 4:**
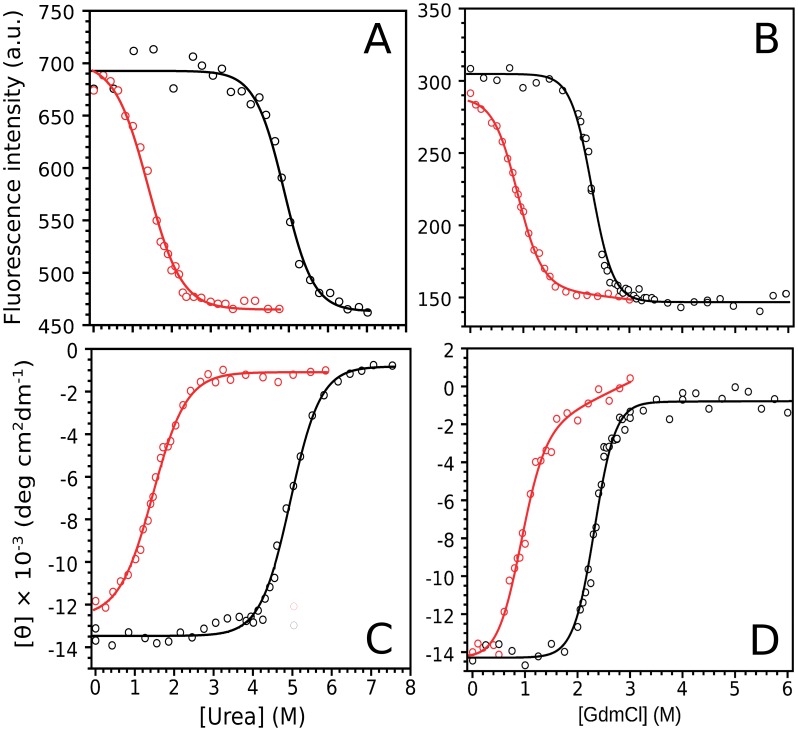
Isothermal equilibrium unfolding experiments followed by tryptophan fluorescence (A and B) and far-UV CD (C and D). Unfolding was induced by urea (A, C) or GdmCl (B, D). Protein variants were incubated with denaturant for 16 h. GdmCl and urea stock solution concentration (8.0 M and 10.0 M) was determined using a standard refractometer. The measurements were carried out at 20°C in 20 mM Tris·HCl, 100 mM NaCl, 1 mM EDTA, pH 7.0. The solid lines represent the nonlinear regression fitting (two-state model, see Materials and Methods).

**Figure 5:**
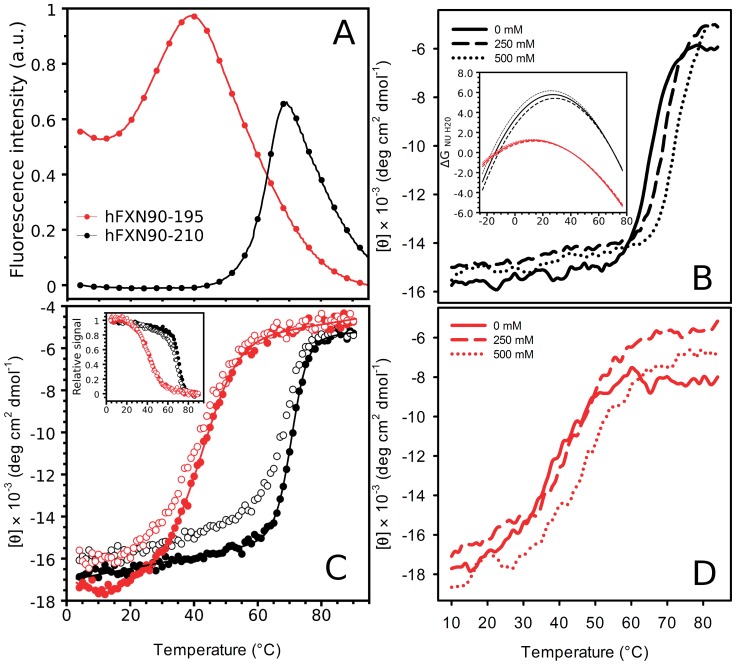
Thermal unfolding of variants hFXN90–210 and hFXN90–195. Unfolding was followed by (A) Sypro-orange fluorescence in 10 mM sodium phosphate, pH 7.0, and by far-UV CD spectroscopy (B, C, and D). For both variants hFXN90–210 and hFXN90–195 (B and D, respectively) unfolding experiments were also conducted in the presence of different salt concentrations (0, 250 and 500 mM NaCl). In (C), the measurements were carried out in 20 mM sodium phosphate, 100 mM NaCl, 0.1 mM EDTA, at pH 7.0. Heating from 4 to 90°C and cooling from 90 to 4°C are shown in filled and open symbols, respectively. The inset in (B) shows for both proteins the dependence of the free energy of unfolding with the temperature for the buffer condition 20 mM sodium phosphate, 100 mM NaCl, 0.1 mM EDTA, pH 7.0. The curves in dotted lines are plotted taking into account the two extremes of the standard deviation of the ?H_NU_ parameter. The inset in (C) shows the CD signals at 220 nm normalized between 0 and 1 to facilitate the comparison between Tm values obtained in unfolding and refolding and reversibility.

Interestingly, hFXN90–195 acquires a native-like fold after refolding *in vitro*. However, residues involved in interactions with CTR form an apolar network that may be a key component of hFXN core. To investigate the effect of protein truncation on the thermodynamic stability of hFXN, we performed equilibrium unfolding experiments followed by far-UV CD and tryptophan fluorescence intensity as probes of secondary and tertiary structures, respectively (Figure 4). Dialysis of chemically unfolded hFXN90–210 and hFXN90–195 showed >95% reversibility. (Figure S3). The equilibrium unfolding curves are well described by a two-state model. Variant hFXN90–195 is significantly destabilized in comparison to variant hFXN90–210, as shown by the differences in ?G^○^_NU H2O_ between these proteins (Table 2). Temperature unfolding was also studied. First, the process was followed by monitoring changes in the fluorescence signal of SYPRO orange dye as it interacts with a protein undergoing thermal unfolding [32], [33], [34]. The T_m_ obtained for hFXN90–195 was 28–30°C whereas for the wild-type protein, the value measured was 64°C (Figure 5A). The lower T_m_ value observed for the truncated variant is compatible with the lower stability measured by chemical unfolding. On the other hand, no dependence of Tm values on protein concentration was observed in the range of 0.16, 0.33 and 0.5 mg/mL (data not shown). It is important to observe that unfolding curves followed by SYPRO orange fluorescence were performed at low ionic strength (see below).

**Table 2:** GdmCl- and Urea-induced Unfolding Parameters for the hFXN Variants.

Two-state Model (N↔U) GdmCl										
hFXN	Fluorescence					Circular Dichroism				
	ΔG°_NU_	m_NU_	C_mNU_	S0_N FL_	S0_U FL_	ΔG°_UN_	m_NU_	C_mNU_	S0_N_	S0_U_
**90–210**	6.7±0.2	2.9	2.3±0.1	304±1	147±1	6.7±0.2	2.9	2.3±0.1	−14.3±0.2	−0.8±0.5
**90–195**	2.2±0.1	2.5	0.8±0.1	288±2	161±3	2.0±0.2	2.5	0.8±0.2	−14.0±0.2	−4.1±0.8
**Two-state Model (N↔ U) Urea**										
**hFXN**	**Fluorescence**					**Circular Dichroism**				
	**ΔG°_NU_**	**m_NU_**	**C_mNU_**	**S0_N FL_**	**S0_U FL_**	**ΔG°_UN_**	**m_NU_**	**C_mNU_**	**S0_N_**	**S0_U_**
**90–210**	7.1±0.1	1.45	4.9±0.1	693±4	461±5	7.2±0.2	1.45	5.0±0.	−13.5±0.2	−0.8±0.2
**90–195**	1.7±0.1	1.25	1.2±0.1	707±6	463±3	1.8±0.1	1.25	1.4±0.1	−12.8±0.2	−1.0±0.1

Parameter m_NU_ is the slope of the linear dependency of free energy of unfolding (ΔG_NU_°) on denaturant concentration and ΔG°_NU H2O_ is ΔG°_NU_ at zero denaturant concentration. In the fittings, we used the predicted m_NU_ values for GdmCl- and urea-induced unfolding considering a globular protein of a given molecular weigtht, 2.9 and 1.45 kcal mol^−1^ M^−1^ for hFXN90–210 and 2.5 and 1.25 kcal mol^−1^ M^−1^ for hFXN90–195 [68]. The units of m_NU_ are kcal mol^−1^ M^−1^. C_m_ and free energy are M and kcal mol^−1^, respectively. CD signals are in [Θ] ×1 10^−3^ (deg cm^2^ dmol^−1^), and fluorescence intensity signals are arbitry units. The parameters were calculated by nonlinear least square fit of the data shown in Figure 4 as described in Materials and Methods.

In addition, the presence of dye binding to the truncated form even at low temperatures would indicate (a) that hFXN90–195 exhibits hydrophobic surfaces, when compared to variant hFXN90–210; (b) a heterogeneous native state ensemble where conformations fluctuate between states exposing binding sites; or (c) the existence of a small fraction of unfolded molecules at low temperatures, when no chaotropic agents are added. The latter is compatible with the lower thermodynamic stability of hFXN90–195 variant and is also in agreement with DLS and NMR experiments mentioned above.

To characterize in detail the temperature unfolding process we performed thermal unfolding experiments followed by far-UV CD (Figure 5B, C and D and Table 3). In agreement with the fluorescence experiments, the variant hFXN90–195 shows the transition at significantly lower T_m_ (T_m_ values are 40.4 and 70.5°C for truncated and full-length hFXN, respectively, Figure 5C and Table 3). In both cases, the unfolding process is reversible as judged by the recovery of the spectroscopic signals upon cooling protein solutions to the starting temperature, 4°C (refolding yields are 96 and 99% for the truncated and full-length variants, respectively, Figure S4A). When proteins were cooled at a lower rate (with data acquisition) 87 and 91% of refolding was observed in the case of hFXN90–195 and hFXN 90–210, respectively (Figure 5B), indicating that, in this case, an aggregation process may compete with refolding. Similar results (95% of refolding at pH 7.0) were observed for hFXN90–210 by Correia and coworkers [29]. In addition, neither a significant variation in the apparent T_m_ in consecutive ramps (Inset in Figure 5C and Figure S4A) nor a dependence of T_m_ value with the heating rate in the range assayed was observed (Figure S4B).

**Table 3:** Temperature-induced Unfolding Parameters for the hFXN Variants.

Two-state Model (N↔U) Temperature						
hFXN	ΔH_NU_(kcal mol^−1^)	T_M_(°C)	ΔC_P NU_(kcal mol^−1^ K^−1^)	ΔG_NU__20 C_(kcal mol^−1^)	ΔG_NU_ _MAX_(kcal mol^−1^)	T _ΔGNU MAX_(°C)
**90–210**	92±2	70.5±0.2	1.9	6.1±0.3	6.2±0.3	25
**90–195**	28±2	40.4±0.6	1.0±0.1	1.1±0.1	1.2±0.1	12

The predicted ΔC_P NU_ values asuming the complete unfolding of the molecules are 1.9 and 1.7 kcal mol^−1^ K^−1^ for hFXN90–210 and for hFXN90–195, respectively. [68] The parameters were calculated by nonlinear least square fit of the data shown in Figure 5B as described in Materials and Methods, using equations 1 and 2. ΔG_NU__20 C_ is the free energy of unfolding at 20°C and ΔG_NU_ _MAX_ is the highest value of free energy.

The dependence of the stability on the NaCl concentration was explored for both variants (Figure 5B and D). The Tm values determined by CD were 36.2, 43.3 and 45.4 (0.0, 250 and 500 mM NaCl, respectively) in the case of the truncated form. On the other hand, values for hFXN90–210 were 66.6, 71.6 and 73.5°C (0.0, 250 and 500 mM NaCl, respectively), suggesting the existence of similar electrostatics contributions in both proteins. We think that the salt effect on the stability of both hFXN variants is a consequence of the shielding of negative charges of Glu and Asp amino acid residues located in the N-terminal (helix α1 and strand β1). This is reinforced by the stabilizing effect produced by mutation of acidic side-chains, observed and documented in detail by Gomes et al [35]. In this case, the mutant D86A/E90A/E93A/D101A/E103A of yeast FXN is stabilized in 2.6 kcal compared to wild-type protein. The thermodynamic stability of hFXN90–210 and hFN90–195 proteins does not depend on pH in the range from pH6.0 to pH8.0 (data not shown) [29]. A similar behavior was also observed in the cases of *E. coli* and yeast FXN variants [30].

We suggest that the lower T_m_ values observed in unfolding experiments of hFXN variants using the SYPRO orange probe, in comparison with the values obtained by CD (ΔT_m_ are ∼6 and 2.4°C, for hFXN90–195 and hFXN90–210, respectively) might be due to an extra destabilization caused by the binding of the dye to the unfolded state of these proteins. In addition, the slight variation in sodium phosphate concentration (10 mM instead of 20 mM) may contribute to this difference.

The results presented in this section confirm that the truncated version, despite being globular and rather compact, displays a decreased thermodynamic stability in comparison with the wild-type hFXN.

Interestingly, in the case of hFXN90–195 a considerable difference in the apparent folding free energy is observed between thermal and chemical unfolding (1.1±0.1 and 1.9±0.2 kcal mol^−1^, respectively). We cannot rule out that the nature of the temperature-unfolded state would be slightly different in comparison to the chemical-induced unfolded state. The ?C_P_ value for this variant (1.0±0.1 kcal mol^−1^ K^−1^) is only 58% of the expected value (1.7 kcal mol^−1^ K^−1^), whereas urea-induced unfolding is well adjusted with a value of m_NU_, the slope of the linear dependence of free energy of unfolding (?G_NU_°) on denaturant concentration, compatible with the ?ASA_NU_ where U is completely unfolded. Thus, this difference might be a consequence of a higher degree of compaction in the case of the temperature-induced unfolded state of hFXN90–195 (particularly at low temperatures, Tm = 40°C).

As mentioned above, the existence of a small fraction of molecules in unfolded conformations (∼10%) in equilibrium with the native state is inferred from the extremely low thermodynamic stability of variant hFXN90–915, in the absence of chaotropic agents when incubated at 20–25°C.

To evaluate this, far-UV CD spectra of both variants were also acquired in the presence of 200 mM Na_2_SO_4_, an osmolyte that modifies the relative stabilities of N and U state, leading to an increase in global stability and compactness [36]. In this experimental condition, we observed a slight increment in the CD signals in the far-UV region for variant hFXN90–195, compatible with the acquisition or stabilization of secondary structure (the relative increment was 11.2±2.1%, Figure S5). On the other hand, in the case of hFXN90–210 the relative increment was significantly lower (4.0±1.6%).

### Dynamics and Flexibility of hFXN

#### hFXN90–195 is more flexible than hFXN90–210

It is well known that proteases require exposure of a specific site and significant backbone flexibility to exert their function. To study the effect of the CTR truncation on the flexibility of hFXN, we performed a limited proteolysis experiment followed by RP-HPLC and RP-HPLC-MS to identify protease-accessible sites. Chymotrypsin protease was selected because 14 aromatic amino acid residues are located along the polypeptide chain (Figure S6) and their distribution enabled us to investigate the dynamics of the backbone at different sites.

**Figure 6:**
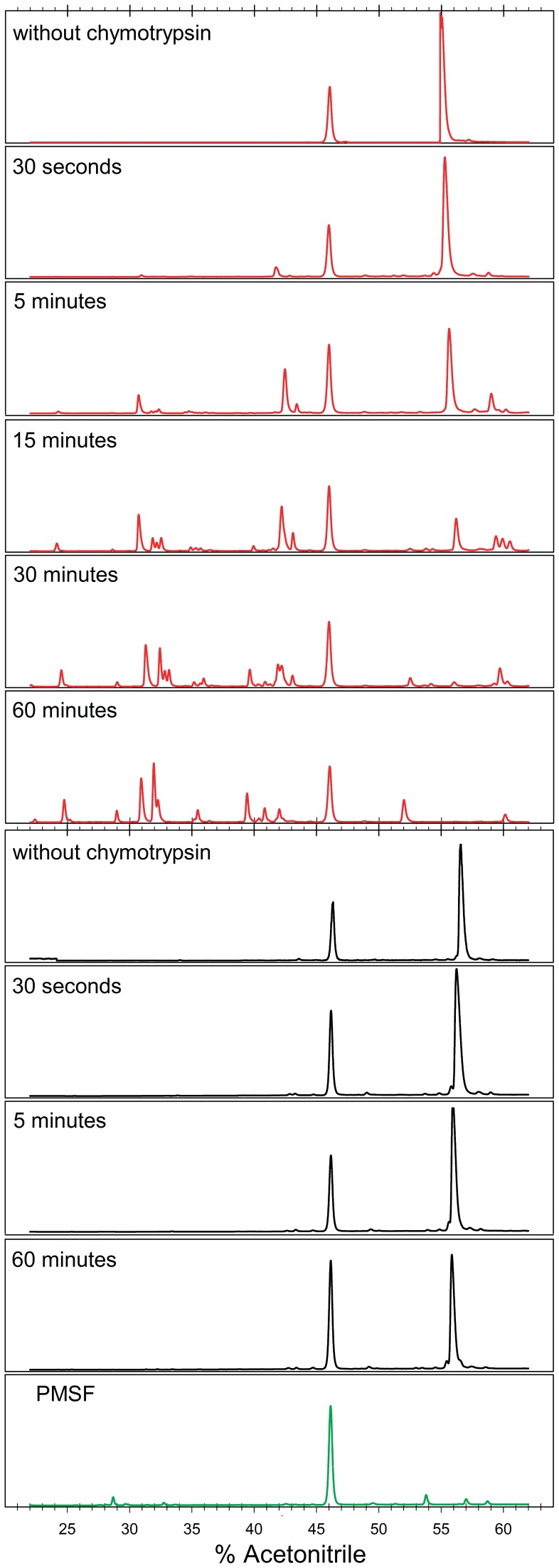
Controlled proteolysis of hFXN90–195. Experiments were followed by RP-HPLC. Chromatograms corresponding to hFXN90–210 and hFXN90–195 are shown in black and red, respectively. Protein concentration was 1.0 mg/mL and chymotrypsin was 1∶200, protease to protein, mass ratio. The reaction was performed at 20°C in buffer 20 mM Tris-HCl, 100 mM NaCl, 0.1 mM EDTA, pH 7.0. The chromatogram corresponding to the solvent plus PMSF is in green.

According to previous reports, hFXN90–210 shows marked protease resistance. [26], [29] After long incubation times with protease (4 h), we detected only one cutting site. This site is located in CTR (residue Y205). More probably, this shows that the flexibility of the region is higher in comparison to the stiffness of the rest of the protein. The truncation of the CTR in the hFXN90–195 variant determines a significant alteration of this behavior. At very short incubation times (between 20 sec to 5 min) and 200∶1 (protein to protease mass ratio, at 25°C), no less than six backbone cuts occurred (Y118, Y143, W155, Y166, and W173 and Y175), as judged by ESI and MALDI mass spectrometry analysis (Figure 6, S6).

Regarding the proteolytic sites observed at short incubation times with protease, sites W155 (located in strand β4) and Y175 (situated in the connector loop between strand β6 and helix α2) are sites partially exposed to the solvent (using PDBID: 1EKG and MOL-MOL software [37]). On the other hand, residues Y143, located in β3 under helix α2 and the CTR, Y166, in strand β5, and W173 situated in strand β6. Therefore, we speculate that these cuts could take place after major conformational changes, including global or local unfolding events.

In particular, residue Y118 is located in loop 1 (connecting helix α1 and strand β1, 18% of solvent accessibility). Interestingly, the analysis of the X-ray structure of hFXN90–210 with COREX/BEST algorithm [38], [39] points to loop 1 (residues D115 to Y123) as the region with the highest probability of undergoing local unfolding (Figure S7). Likewise, the Protein Frustratometer algorithm [40] points out that the CTR forms a network of minimally frustrated interactions with α1 and α2 (Figure S8).

Despite the similarities observed in secondary and tertiary structure and hydrodynamic behavior, the results of proteolysis experiments might be explained by a difference in flexibility between full-length and the truncated variants.

**Figure 7:**
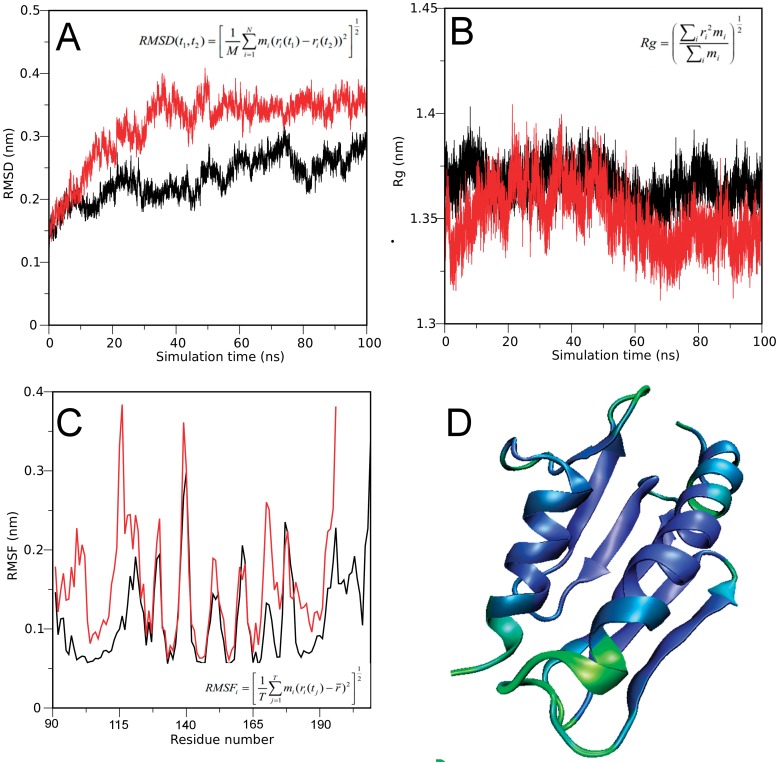
Explicit-solvent all atom simulation of full-length (black lines) and truncated hFXN (red lines). (A) Root Mean Square Deviations (RMSD) and (B) Radius of gyration (Rg) were computed along the 100 ns of simulation. (C) The RMSF values per residue were computed from the equilibrated part of the trajectory, which was from 3 ns of simulation. (D) hFXN90–195 colored by the degree of flexibility of the backbone. Blue and green represents the least and most flexible regions of the backbone, respectively.

To characterize the effect of the CTR truncation on hFXN dynamics, we performed molecular dynamics simulations (MDS). We explored fast conformational dynamics with explicit solvent in classical all atom simulations using empirical force fields. The starting model for hFXN90–210 was PDB ID = 1EKG, and we modeled hFXN90–195 truncating the CTR of the protein. Each variant was subjected to a 100 ns run. An initial inspection shows that both proteins remain native-like, so that truncation does not produce large conformational alterations in this timescale. In this way, RMSD and *R*g values corresponding to the ensemble of conformations of the truncated variant are compatible with those observed for wild-type hFXN, and the general topology of the protein remains somehow stable during the simulation time (Figure 7 A and B). However, when hFXN90–195 fluctuations are cautiously examined, the region involving loop 1 (D115 to Y123) is detected as one of the regions with the highest RMSF (Figure 7 C and D). The difference between RMSF values in this region is observed in different time intervals along the simulation (data not shown). This result, together with chymotrypsin experiments, reinforces the idea of an enhanced flexibility in variant hFXN90–195.

#### Structure-based model simulations show that hFXN visits two native-like isoenergetic conformations and that deletion of residues 196–210 affects folding kinetics

**Figure 8:**
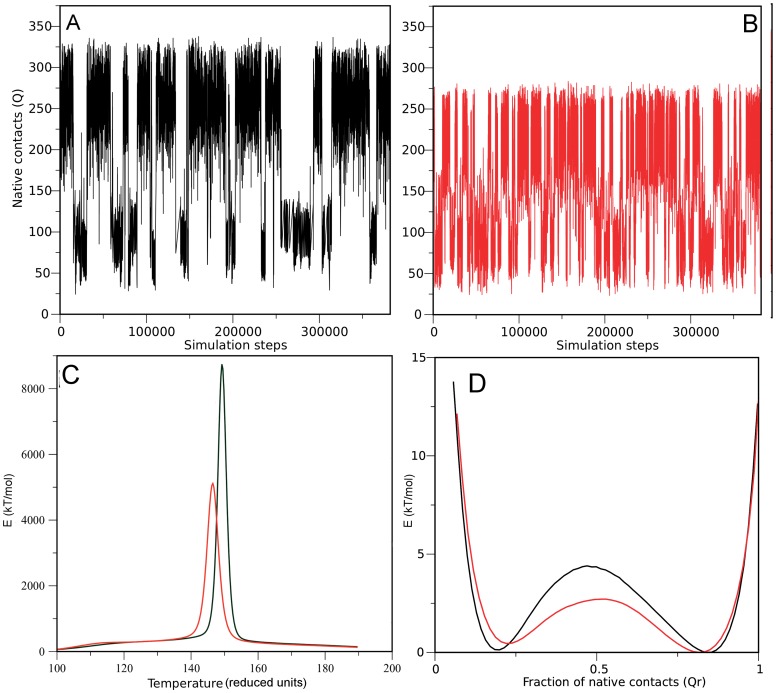
Structure-based model simulations of hFXN90–210 (A) and hFXN90–195 (B). Number of native contacts formed (Q) as a function of the number of steps of the simulations. The observed transitions represent the folded to unfolded state conformational change. (C) Calorific capacity at constant volume for both variants. The peak represents the folding/unfolding transition. (D) Energy profile for both variants as a function of the relative native contacts at the melting temperature of each variant (1 represents all the native contacts formed, while 0 represents none of the contacts formed). The minima represents the folded (Q∼0.8) and unfolded (Q∼0.2) states, while the energy barrier (Q∼0.5) represents the transition barrier for this reaction.

In order to dissect the contribution of simple topological constraints in the folding dynamics of the hFXN 90–210 and fragment hFXN90–195, we analyzed the folding behavior of these proteins on perfectly funneled energy landscapes [41]. We performed simulations with structure-based models, in which all the sequence information is removed and the average native structure is the sole input [41]. We derived the potential from the PDBID = 1EKG as described in methods, and performed several MD runs to explore the phase-space. Typical trajectories are shown in Figure 8A and B. Qualitatively, both the hFXN90–210 and hFXN90–195 spend most of the time in either folded (high Q) or unfolded (low Q) ensembles, with no obvious stable intermediate state. Notably, transitions of the shorter protein occur much more frequently (Figure 8B), than the larger one (Figure 8A). To quantify the topological effect of deleting the CTR, we used weighted histogram analysis method WHAM [42], [43] to extract the mean thermodynamic parameters. Both proteins show a single peak in the heat capacity as a function of temperature (Figure 8A). hFXN90–210 displays a sharper peak at a slightly higher temperature, showing that the deletion of the CTR not only destabilizes the native fold but also affects the cooperativity of the main folding transition.

The free energy profiles of the proteins at their respective T_m_ show two minima, which we ascribe to the folded (high Q) and unfolded (low Q) structural ensembles (Figure 8D). A smooth free energy barrier at Q∼0.5 separates these states, suggesting that both proteins fold via a two-state like mechanism. Notably, the barrier height differs in about 2.3 kT/mol (∼50%), showing that the deletion of CTR strongly affects the overall folding kinetics of the hFXN fold, as was apparent in the raw traces (Figures 8A and 8B).

**Figure 9:**
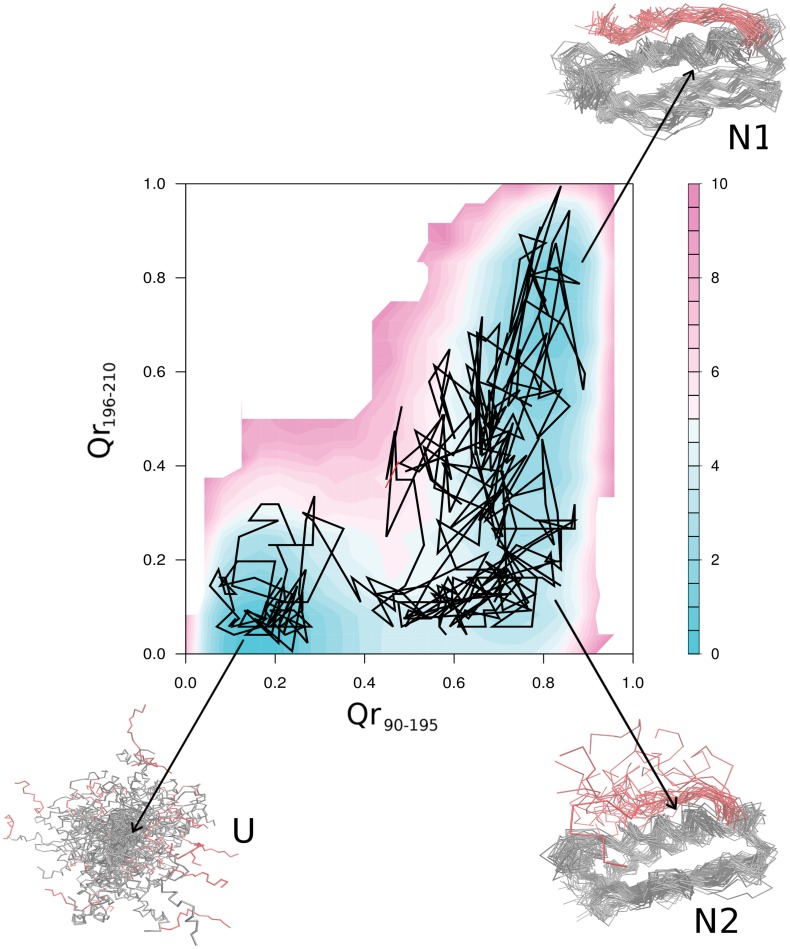
Contour plot of the contacts between CTR and the rest of the protein. The color represents the log of the probability of the intersection event. The black line represents a representative fraction of the trajectory. In addition, the superimposition of hFXN conformations corresponding to clusters N1, N2 and U populated during the folding dynamics are shown. Residues 90–195 and 195–210 are shown in grey and red, respectively.

The native ensemble for hFXN90–210 is broad, forming between 200 and 300 contacts at T_m_, with a global RMSD of ∼0.3 nm. When the thermodynamics of the contacts of the CTR region is evaluated, it is apparent these undergo a concerted change in the interaction with the hFXN core, being either formed or not formed (Figure S9). This transition peaks at a lower temperature than the core transition (Figure S9C), thus, at the T_m_ of the hFXN90–210, the CTR is stabilized to unbound conformations (Figure S9A). This suggests that the native ensemble of hFXN populates at least to distinguishable substates, N1 and N2 (Figure 9). In N2 the main core of FXN is folded, but most of the tertiary interactions involving the CTR are not formed, consolidating only in N1 substate. In these free-energy representations it appears that N2 is an obligatory intermediate connecting N1 and U, suggesting that unfolding of hFXN starts with unbinding of the CTR (Figure 9). Concomitantly, hFXN would fold *via* nucleating at the core and not at the CTR. To directly quantify this we computed the probability of individual contact formation for the transition state ensemble (TSE) separating U and N1. We observe that the TSE has the overall topology of the hFXN fold (Figure S10). Thus the CTR region appears to stabilize the native ensemble by acting as a ‘lock’ to the core region. On the other hand, CTR affects the folding kinetics of the core, even when not directly participating in the TSE. Preliminary experiments show that unfolding kinetics of hFXN90–195 is indeed substantially faster than the full-length protein (Figure S11). In this context, we suggest that CTR may act producing a slowdown in folding reaction, probably by called “backtracking” [44], [45], [46].

## Discussion

A glance at the structure of hFXN points to the C-terminal region (CTR) as a distinctive part of the protein in terms of the FXN conformation. The CTR does not form a typical secondary structure element (neither an α-helix nor a β-strand), nevertheless, it establishes a large number of long-range interactions with both N- and C-terminal α-helices (Figure S8). It appears that this part of the protein is not crucial for the acquisition of FXN fold as yeast FXN does not have CTR, and *E. coli* FXN has a shorter one compared to the hFXN. It was previously reported that, the stability of yeast FXN (yFXN) is largely diminished in comparison to the hFXN variant. The T_m_ and ΔG°_NU H20_ values are 40°C and 1.5 kcal/mol for yFXN and 70.5°C and 6–7 kcal/mol for hFXN, indicating that the CTR would be related to the stability of FXN fold [30], [31]. Interestingly, the engineering of a CTR increases the thermodynamic stability to the yFXN variant [30]. Nevertheless, it was not possible to determine if these differences in thermodynamic stability were originated from the absence of CTR or due to other existing sequence dissimilarities.

Previous attempts to express a shorter version of hFXN were not successful. When a variant, which lacks most of the CTR (hFXN91–198), was expressed in *E. coli*, it was persistently found in inclusion bodies [30].

Here, by tuning the sequence deletion to hFXN90–195, we successfully obtained enough recombinant protein to perform experiments and derive biophysical parameters to characterize the protein.

Classical spectroscopic signatures show that the conformation of hFXN90–195 is native-like (Figure 2). However, wild-type hFXN displays both higher chemical shift dispersion (Figure 3) and enhanced near-UV CD bands (Figure 2) when compared to the truncated variant, a result compatible with a loss of tertiary packing. On the other hand, the CTR deletion strongly destabilizes the overall fold, as seen when followed by both temperature (T_m_ values are 40.4 and 70.5°C for hFXN90–195 and hFXN 90–210, respectively) and chemical denaturation (ΔG°_NU_ values are ∼1–2 and ∼6–7 for hFXN90–195 and hFXN 90–210). Interestingly, the difference between the fitted ΔC_P NU_ values, obtained from temperature denaturations indicates that, in the absence of chaotropic agents (like urea or GdmCl), hFXN90–195 may unfold with a smaller solvent accessible surface area difference (ΔSASA_NU_) than hFXN90–210. Similar results were previously described for the NCA mutant of staphylococcal nuclease protein [47]. The experimental determination of ΔC_P_ values by DSC will help in the future with this.

Topology is known to be a main determining factor of folding transitions [48]. To prove whether the folding behavior observed for the CTR deletion can be attributed to topological effects, we performed folding simulations of perfectly funneled energy landscapes [41]. We observed that the truncated variant is less stable displays a broader temperature transition than the hFXN90–210 counterpart (Figure 8C), qualitatively matching the experimental observations discussed above. Moreover, we identified that CTR residues interact with the 90–195 core in a concerted way, behaving as a folding element that “snaps” onto the rest of the protein (Figure 9). The broad native ensemble of hFXN can be decomposed into substates that either include or do not include CTR consolidation (Figure 9). Unbinding of the CTR promotes unfolding of the rest of the protein, which folds by nucleating at the 90–195 region (Figures S9 and S10). Even when not participating in the main transition state ensemble (TSE), CTR is predicted to slow down the transitions (Figure 8). Preliminary experiments suggest that unfolding kinetics of hFXN90–195 is indeed substantially faster than the full-length protein (Figure S11). Thus, the main differences in the folding dynamics of the hFXN variants can be attributed to topological effects of the long-range interactions of the CTR, that stabilizes both thermodynamically and kinetically the 90–195 region.

All-atom explicit solvent simulations reveal that a more flexible backbone would be one of the main differences between the variants as analyzed in the fast timescale regime. This occurs at specific sites along the polypeptide region including the connector loop 1 (D115 to Y123). In agreement with this, COREX/BEST [38], [39] results show that this region has the highest probability of local unfolding. In accordance, the “Protein Frustratometer” suggests this region is enriched in highly conflicting interactions (Figure S8) [40]. Proteolysis is a very attractive tool to map high-energy conformations [49], [50] While the hFXN90–210 variant is resistant, in the truncated variant six chymotrypsin cleavage sites are detected in the first 5 min of the proteolysis reaction. Both transient local unfolding and/or the presence of unfolded molecules in solution, as a consequence of the low thermodynamic stability of hFXN90–195, may contribute to this behavior.

We also considered the possibility that changes in the dynamics of CTR may alter the dynamics of the hFXN β-sheet. In this regard, W155 is one of the six porteolytic sites that we observed after short incubation with chymotrypsin, in the case of the truncated form. Thus, we speculate that changes in the β-sheet dynamics could produce a substantial modification of hFXN molecular surface. Consequentially, alterations of the CTR would alter the binding and activation of the SDUF protein complex involved in iron metabolism and Fe−S cluster biosynthesis [28], tuning its biological function.

Although variant hFXN90–210 is markedly resistant to protease, a peptide involving the last six residues of the CTR is removed by chymotrypsin, suggesting that part of the CTR of hFXN is indeed quite mobile, in contrast to the hFXN core, which is in agreement with NMR results (Figure 3) and the structure-based simulations (Figure 9). This is reflected by the incremented internal motions at the C-terminus on the ns- and ps timescale [26], and by the larger RMSD of the solution bundle observed in the NMR structure (PDBID = 1LY7). [27] NMR experiments are underway to further explore the effect of specific point mutations and truncation of the CTR on backbone dynamics.

Two point mutations located in the CTR have been found in FRDA patients: S202C and L198R [51]. The latter, introduces a positive charge in the apolar interaction surface between the CTR and residues from both helices α1 and α2. We suggest that these mutations could produce changes in the dynamics of the CTR, probably influencing the stability and/or dynamics of the FXN core, as it happens in the case of variant hFXN90–195 due to the absence of the CTR. In this fashion, FOLD-X algorithm [52], [53] predicts that L198, L200 and L203 form strongly stabilizing interactions (Figure S7).

Here we propose that the local unfolding of CTR may be the initializing step for the global rearranging of hFXN. The CTR seems to play a relevant role in the kinetic stabilization of hFXN fold acting as a conformational “lock” (Figures 8, 9, S9 and S10). In this model, the magnitude of the barrier from native to the unfolded state may be largely perturbed by point mutations like L198R. The functional deficiency of hFXN L198R mutant could be explained by destabilization of the CTR-α-helical interactions.

In this regard, hFXN90–195 shows deficiency in protein-iron interaction, producing insoluble particles in the presence of iron (Figure S12). A similar behavior was observed for mutants I154F and W155R that precipitate upon iron interaction [26].

Taking into account the preponderant role of the CTR in stabilizing hFXN molecule through the consolidation of α-helical unit, directly affecting the population of different native substates in solution, we infer that the specific mutation of the helical elements may also have consequences in kinetic or thermodynamic stability, folding dynamics, and biological function. Suggestively, the mutation of residue L182 which mediates apolar interactions between C-terminal helix and β-sheet is also associated to FRDA indicating a probable relation between L182F mutation and the loss of the biological function of the hFXN [54]. C-terminal helix is amphipathic and probably locally stabilized by a leucine tandem L182, L185, L186, L190 and L194. [55] The AGADIR algorithm [52], [56] predicts the mutation L182F would reduce the propensity of the α-helical element from 13% to 6%. In addition, tertiary packing effects should be taken into account as a consequence of the presence of a more bulky side-chain.

### Conclusion

The CTR is a crucial element in the stabilization of hFXN. Moreover, the presence of this stretch of residues enables this macromolecule to smoothly modulate its stability and dynamics. These motions may play a clue role in the protein function given that the existence of point mutations in this region leads to Friedrich’s Ataxia syndrome.

## Materials and Methods

### Protein Expression and Purification

Human frataxin cDNA was kindly provided by Dr. Hèléne Puccio from IGBMC (Strasbourg, France). Primer sets Fw_210_: tttaagaaggagatatacatatgctagatgagaccacctatgaa Rev_210_: gcatggatcctcaagcatcttttccggaataggc and Fw_195_: tttaagaaggagatatacatatgcta gatgagaccacctatgaa, Rev_195_: gcatggatcctcattttaaggctttagtgagctct where used to amplify the ORFs corresponding to the variants hFXN90–210 and hFXN90–195 respectively. The PCR products were sub–cloned into a pET9b plasmid vector, and the identity of the inserts was confirmed by DNA sequencing.

Bacteria cultures (*E. coli* BL21 (DE3), 2–3 L Terrific Broth, pH 7.2) were grown at 37°C and 280 rpm. Protein expression was induced at DO = 1.0 by addition of 1.0 mM IPTG. After induction for 3.5 hours, bacteria were centrifuged at 6,000 rpm and the pellet was stored at –20°C until cell disruption with French press. Soluble and insoluble fractions were separated by centrifugation at 10,000 rpm (30 min).

For the purification of hFXN90–210, the soluble fraction was incubated with 10 mM EDTA and carefully loaded onto an ion exchange chromatography (DEAE DE52 matrix), and eluted with a 300 mL linear gradient, from 0.0 to 1.0 M NaCl, in buffer 20 mM Tris·HCl, 1 mM EDTA, pH 7.0. Subsequently, fractions (identified by SDS-PAGE) with FXN were loaded onto preparative Sephadex G–100 column (SEC, 93 cm × 2.7 cm), previously equilibrated with buffer 20 mM Tris·HCl, 100 mM NaCl, 1 mM EDTA, pH 7.0. This yields >95% pure hFXN as confirmed by mass spectrometry (ESI-MS) (theoretical molecular mass value: 13,605.1 Da, considering the N-terminal methionine residue); hFXN90–210 concentration was determined spectroscopically using an extinction coefficient ε_280nm_ = 26,930 M^–1^ cm^–1^ (1 mg/mL protein solution represents Abs_280nm_ = 2.00).

The truncated variant hFXN90–195 was expressed according to the same protocol, but purified from the insoluble fraction of the lysate. It was solubilized at room temperature from the inclusion bodies (IB) by incubating with 3.0 M urea. Interestingly, this treatment leads to the solubilization of the recombinant protein, whereas other proteins were solubilized only after incubation with 6.0 M urea. Next, a refolding step was performed by dialysis of the fraction against 20 mM Tris·HCl, 100 mM NaCl, 1.0 mM EDTA, pH 7.0. Finally, protein purification was performed, in identical conditions to the procedure previously described for hFXN90–210. Mass was corroborated by ESI-MS (theoretical molecular mass value: 11,923.2 Da); the extinction coefficient used for this variant was ε_280nm_ = 25,440 M^–1^ cm^–1^ (1 mg/mL protein solution represents Abs_280nm_ = 2.13).

### Fluorescence Measurements

Steady–state fluorescence measurements were performed in a Jasco FP–6500 spectrofluorometer operating in the ratio mode and equipped with a thermostated cell holder set at 20°C. To this end, a 1.0 cm path length cell sealed with a Teflon cap was used. When the intrinsic fluorescence of proteins was measured, excitation wavelength was 295 nm and emission data were collected in the range of 305–450 nm. The spectral slit–width was set to 3 nm for both monochromators.

### Circular Dichroism Spectroscopy

Ellipticity of protein samples was evaluated using a Jasco 810 spectropolarimeter calibrated with (+) 10-camphorsulphonic acid. Far–UV CD spectra were recorded in the range between 185 and 250 nm, protein concentration was 10 µM, and a cell of 0.1 cm path length was used. For near–UV CD spectra, the wavelength range was 240–340 nm, protein concentration was 20 µM, and the path length was 1.0 cm. In all cases, data was acquired at a scan speed of 20 nm min^−1^ and at least 3 scans were averaged for each sample. Blank scans were subtracted from the spectra and values of ellipticity were expressed in units of deg cm^2^ dmol^–1^, unless expressed otherwise in the text.

### NMR Spectroscopy

Samples for NMR experiments contained 0.5 mM hFXN90–195 or 0.8 mM hFXN90–210, 20 mM Tris·HCl pH 7.0, 100 mM NaCl, 1 mM EDTA, in a solution of 90% H_2_O/10% D_2_O. The NMR experiments were performed from 290 to 315 K on a Bruker 600 MHz Avance III spectrometer equipped with a 5 mm triple resonance cryoprobe incorporating shielded z-axis gradient coils. The NMR data were processed and analyzed using Tospsin 3.0 software.

### Limited Proteolysis

hFXN variants (1 mg/mL) were incubated with chymotrypsin at mass ratios 1∶50, 1∶100, 1∶150 and 1∶200 (protease:protein), at different temperatures (4, 25 and 55°C) in buffer 20 mM Tris·HCl 100 mM NaCl, 1 mM EDTA, pH 7.0. Aliquots were separated at different times (0, 5, 10, 15, 30, 45 min, and 1, 1.5, 2, 3, 5, 20 h) and the reaction was immediately stopped by addition of 0.2% TFA and 1.0 mM PMSF. Samples were kept at −70°C until analysis by SDS–PAGE and RP–HPLC followed by MALDI or by ESI–MS.

### Protein Unfolding Experiments

Isothermal unfolding experiments were carried out incubating the hFXN variants with the appropriate concentration of the chaotropic agent in buffer solution (20 mM Tris·HCl 100 mM NaCl, 1 mM EDTA, pH 7.0) for 16 h at room temperature. All measurements were done at 20°C. The process was followed by far-UV CD recording and tryptophan fluorescence measurement. In order to calculate thermodynamic parameters, a two–state unfolding mechanism was assumed, where only native (N) and unfolded (U) conformations exist at equilibrium. Data processing was performed according to Santoro and Bolen [57], [58], [59]. Denaturant concentrations were determined using a standard refractometer.

Thermal unfolding was monitored by changes in the fluorescence signal of SYPRO orange dye by heating the holder from 0 to 95°C at a rate of 1°C min^−1^ [32], [33], [34]. The experiment was performed in a real time PCR system (Biorad). The excitation and the emission ranges were 470–500 and 540–700 nm, respectively. Protein concentration was 0.16 mg/mL and buffer was 10 mM sodium phosphate, pH 7.0. It is believed that the fluorescence signal is quenched in the aqueous environment, but becomes unquenched when binding the apolar core of the protein upon unfolding.

In addition, unfolding transitions as a function of temperature were monitored by the CD signal at 220 nm. Experiments were carried out in 20 mM sodium phosphate, 100 mM NaCl at pH 7.0. The protein concentration was 7.0 µM, and a 1.0 cm cell path length was used. Temperature was varied from 0 to 95°C, at a rate of 1°C min^−1^, and the melting curve was sampled at 1.0°C intervals. To extract the thermodynamic parameters the following model was fitted to the data: 
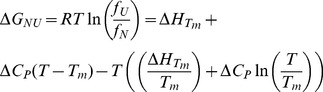
(1)

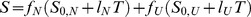
(2)where *f*_U_ and *f*_N_ are the unfolded and folded fractions at equilibrium, respectively; *Tm* is the temperature at which *f*_U_ = *f*_N_; *S* is the observed CD signal; *S*_0,N_ and *S*_0,U_ are the intrinsic CD signals for the native and unfolded states, respectively; *l*_N_ and *l*_U_ are the slopes of the pre- and post- transition regions, respectively, assuming a linear dependence of *S*_N_ and *S*_U_ with temperature.

The reversibility of the chemical unfolding reactions for both hFXN variants was verified by dialysis. Proteins were incubated for three hours with urea or GdmCl at different concentrations (at room temperature) to ensure equilibrium conditions and to minimize chemical modifications (in particular, in the case of urea solutions long incubations were avoided). Next, proteins were extensively dialyzed (overnight at 4°C) against buffer 20 mM Tris-HCl, 100 mM NaCl, pH 7.0. After dialysis, more than 95% of the protein was recovered as evidenced by the protein concentration measured by UV spectroscopy. No significant aggregates were observed, as judged by the absence of light scattering. Tryptophan fluorescence spectra of proteins at different denaturant concentrations were acquired before and after dialysis. More importantly, in all cases the spectra were similar in intensity and λ_MAX_.

The reversibility in each temperature unfolding experiment was investigated by comparing the initial value of CD signal at 220 nm with the signal value recovered after cooling the protein solution that underwent unfolding. In addition, the CD signal at 220 nm was also acquired at a rate of 1°C min^−1^ from 95 to 0°C.

### Hydrodynamic Behavior

Changes in hydrodynamic volumes were monitored by chromatography on a SEC–HPLC system equipped with a 280 nm UV detector and a Superose12, or a Superdex S–200 HR 10/30 columns (Pharmacia Biotech, Sweden) equilibrated at room temperature in buffer 20 mM Tris-HCl, 100 mM NaCl, 1 mM EDTA, pH 7.0. This type of chromatographic resin lets us examine the presence of soluble aggregates in the samples. The flow rate was 0.2 to 0.5 mL min^−1^ and the injection volume was 100–200 µL. Samples were centrifuged at 14,000 rpm before loading onto the column, which was previously calibrated with appropriate molecular weight markers of known Stokes radii (R_S_) [60].

Dynamic light scattering analysis was also performed to investigate the hydrodynamic behavior and quaternary arrangement of hFXN90–195 variant. In this case, 1 mg/mL (buffer 20 mM Tris-HCl, 100 mM NaCl, 1 mM EDTA, pH 7.0) was analyzed in a standalone dynamic light scattering instrument (DynaPro NanoStar from Wyatt Technology). Experiments were performed in batch mode at 25°C. Samples of 50–100 µL were filtered by 0.22 µm and centrifuged for 20 min at 10000 rpm at 4.0°C. Size distribution by mass was determined using isotropic spheres as the model. Polydispersity (%Pd), is equal to the standard deviation of the distribution from the mean value weighted by its mass fraction divided by the mean R_S_ and multiplied by 100.

In addition, analytical ultracentrifugation experiments (AUC) were also performed to gain a better characterization of both protein variants in aqueous solution. All AUC experiments were performed on a Beckman Coulter XL-I analytical ultracentrifuge. Sedimentation velocity (SV) experiments of solutions were performed at 20 °C, at a rotor speed of 42,000 rpm using the 8-hole ANTi-50 rotor. Cells were equipped with sapphire windows. Titane double sector centerpieces from Nanolytics Inc. were used. Cells were filled, for centerpieces with 1.2 and 0.3 cm optical path, with 400 and 100 µl, respectively, of sample and solvent reference. SV profiles were acquired overnight, using absorbance optics, at intervals of 13 min for each cell. In order to study non ideality effects of the solution, various protein concentrations were investigated (1.0, 0.5, and 0.25 mg/mL) and the sedimentation and diffusion coefficients at infinite dilution, s0 and D0, were derived from the linear approximations. Density and viscosity of the buffer, which are required for the analysis, were calculated with SEDNTERP software, from John Philo (http://www.jphilo.mailway.com/). Analyses of SV experiments were made using the continuous distribution c(s) and the non-interacting species model analysis of SEDFIT software from P. Schuck (http://www.analyticalultracentrifugation.com). SEDFIT software uses a numerical solution of the Lamm equation and incorporates the possibility of accounting for the systematic noise of the experimental data. Buffer was 10 mM Tris-HCl, 100 mM NaCl, pH 7.0.

### Spectophotometric Determination of Iron

Iron concentration was determined using a colorimetric method based on the coordination of Fe^2+^ by 1,10–phenanthroline [61]. Briefly, 1,10–phenanthroline was prepared in 0.1 N HCl, and a standard iron solution (18 mM) was prepared in 0.47 mM sulfuric acid for calibration curves. A volume of 100 µL of ascorbic acid (10%) plus 100 µL of sodium citrate (10%) was mixed with the sample (up to 700 µL) and water (700 µL minus the volume of sample). After that, 100 µL of the 1, 10–phenanthroline solution were added and remixed. Following an incubation of 15 min, the sample was centrifuged in a 1.5 mL tube at maximum speed for 5 min. This centrifugation step is important as protein aggregates may disperse light, generate scattering in the sample and thus lead to incorrect iron determinations. Next, absorbance at 512 nm was read. Routinely blanks were included in the measurements.

### Molecular Dynamics Simulations. Simulation Details

#### Explicit-Solvent all atom simulations

To investigate the fast conformational dynamics of hFXN and hFXN90–195 (in the range of ps to ns), we carried out simulations with GROMACS 4.5.4 and GROMOS 53a6 force field [62]. In all cases, the initial structures were generated from the coordinates of the crystallographic structure PDB ID: 1EKG. The structure of each protein was embedded in a dodecahedral periodic cell with a minimum distance of 0.9 nm between the protein atoms and the cell limits. Both structures were solvated with SPC (simple point charge) water molecules [63]. Sodium and chloride ions were added up to 150 mM salt concentration. One thousand steps of energy minimization were performed. After that, 10000 steps of protein position restrained simulations were carried out to equilibrate water molecules. A canonical ensemble simulation (N.V.T.) using Berendsen thermostat of 120 ps was perform at 300 K and tau = 1 ps^−1^. Later a microcanonical (N.P.T.) simulation using a Berendsen thermostat 120 ps at 300 K and tau = 1 was performed. Finally, 500 ps of simulation applying a restraint to alpha carbons of 25 kJ/mol. The resulting structures were the starting points for the production simulations. For restrained and non-restrained production simulations Nose-Hoover thermostat was used for temperature coupling, while Parrinello-Rhaman thermostat was used for pressure coupling. In all cases, long-range interactions were computed according to the particle mesh Ewald method.

#### Structure-based simulations

To investigate the folding of hFXN in perfectly funneled energy landscapes, structure-based simulations of hFXN were performed [41], [64]. Briefly, each residue is represented by a single bead centered in its Cα position, and adjacent beads are strung together into a polymer chain by means of a potential encoding bond length and angle constraints. The secondary structure is encoded in the dihedral angle potential and the non-bonded (native contact) potential. The interaction energy V for a given protein conformation Γ is given by: 
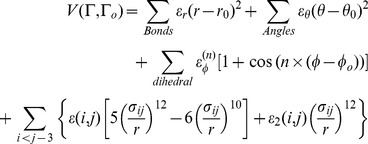
(3)


An interaction between two residues (i, j) exists if the distance between the Cα atoms of the residues is in the range of 4.0 to 6.0 Å.

Native pairs of residues with a distance of *j* ≤ *i*+3 are discarded from the native map as any three or four subsequent residues are already interacting in the angle and dihedral terms [41]. In equation (3), r, θ, and Φ stand for the i*th* virtual bond length between i*th* and (i+1)*th* residue, the virtual bond angle between (i–1)*th* and i*th* bonds, and the virtual dihedral angle around the ith bond, respectively. The parameters r_o_, θ_o_, and Φ_o_ stand for the corresponding variables in the native structure. K_r_, K_θ_, K_Φ_ weigh the relative strength of each kind of interaction entering the energy and they are taken to be K_r_ = 100ε, K_θ_ = 20ε, K_Φ_(1) = ε and K_Φ_(3) = 0.5ε. All native contacts are equally weighted in a 10–12 Lennard-Jones potential. We used the simulation package GROMACS 4.5.4 [65] and the topology, structure, and contact map inputs were calculated using the SMOG server at http://smog.ucsd.edu [64].

A contact is considered to be formed if the distance between the Cα atoms is shorter than γ times their native distance r_0ij_. In this work we used γ = 1.2. Several constant temperature runs were performed and results analyzed by the weighted histogram analysis method (WHAM) [42], [43], using Q (fraction of native contacts) as the main reaction coordinate [66].

## Supporting Information

Figure S1Analytical ultracentrifugation. Sedimentation velocity of hFXN90–195 (left panels) and hFXN90–210 (right panels) at 42000 rpm and 20°C, in 10 mM Tris-HCl, 100 mM NaCl, pH 7.0. (A) Selection of raw data for both proteins at 1 mg/mL. (B) Superposition of experimental (dots) and fitted (continuous line) profiles corrected for all systematic noise for both variants at 1 mg/mL. The last profiles correspond to 16 h of sedimentation. The fit was obtained from the c(s) analysis of the SEDFIT program. For both proteins, the Lamm equation was simulated for 300 particles in the ranges (0.4 S, 15 S) and (0.4 S, 4 S), with a partial specific volume 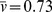 mL g^−1^ and a frictional ratio f/f_min_ = 1.25 (which corresponds to a globular, usually hydrated, macromolecule). (C) Superposition of the differences between the experimental and fitted curves. (D) Corresponding c(s) distribution in the range 0.4–15 S for both variants at 1 mg/mL. The signal was normalized to 1 cm optical path length. (E) Superposition of the c(s) distributions for different concentrations of both proteins in the range 0.4–4 S, corresponding to more than 96% of the total signal. The signal was normalized to 1 cm optical path length.(TIF)

Figure S2 Size distribution of hFXN variants as determined by DLS. Experiments were performed in batch mode at 25°C. Samples of 50–100 µL were filtered by 0.22 µm and centrifuged for 20 min at 10000 rpm at 4.0°C. Size distribution by mass was determined using isotropic spheres as the model. Distributions for hFXN90–210 and hFXN90–195 are shown in black and red, respectively. Proteins (at 1 mg/mL in buffer 20 mM Tris-HCl, 100 mM NaCl, 1 mM EDTA, pH 7.0) were analyzed in a standalone dynamic light scattering instrument (DynaPro NanoStar from Wyatt Technology).
(TIF)

Figure S3 Reversibility of the chemical unfolding reactions for hFXN variants. Reversibility of hFXN90–210 (A, B, C and D) and hFXN90–195 (E, F, G and H) was verified by dialysis. Proteins were incubated for three hours with urea or GdmCl at different concentrations, at room temperature, to ensure equilibrium conditions and to minimize chemical modifications (left panels). Next, proteins were extensively dialyzed (overnight at 4°C) against buffer 20 mM Tris-HCl, 100 mM NaCl, pH 7.0 (the final concentrations of denaturant are detailed in the plots, right panels). Tryptophan fluorescence spectra of proteins at different denaturant concentrations were acquired before (left panel) and after dialysis (right panel). More importantly, in all cases the 5 spectra were similar in intensity and λ_MAX_. After dialysis, more than 95% of the protein was recovered as evidenced by the protein concentration measured by UV spectroscopy. No significant aggregates were observed, as judged by the absence of light scattering.
(TIF)

Figure S4 Reversibility of the temperature unfolding reactions for hFXN variants. (A) Transitions were followed by far-UV CD from 4 to 60°C and 4 to 80°C for hFXN90–195 and hFXN90–210, respectively. When proteins reach the highest temperature they were cooled to 4°C in a fast way, without control of the cooling rate. The signal recovery was 96%, and 99% for hFXN90–195 and hFXN90–210, respectively. The superposition of the consecutive unfolding curves (scan (circles), rescan (squares) and the starting points of the re-rescan (triangles)) are shown for hFXN90–195 and hFXN90–210 in red and black, respectively. The rate was 1°C/min. (B) Unfolding curves for each variant were performed at two different rates = 1.0°C min^−1^ and 0.5°C min^−1^, filled and empty symbols, respectively.
(TIF)

Figure S5 Effect of sodium sulfate on the hFXN90–195 conformation. Far-UV CD spectra of hFXN90–210 (gray) and hFXN90–195 (orange) were acquired in the presence of 200 mM Na_2_SO_4_ or in the absence of the osmolyte (FXN90–210 in black, and hFXN90–195 in red). In addition, spectra of the unfolded states of both proteins were acquired in the presence of 5.0 M GdmCl. The inset shows the CD signal difference (%) upon Na_2_SO_4_ addition. Buffer was 20 mM sodium phosphate, 100 mM NaCl, pH 7.0 and the experiment was performed at 25°C.
(TIF)

Figure S6 Proteolytic sites observed between 20 s and 5 min in hFXN90–195 (red) and hFXN90–210 (blue), respectively. The other aromatic residues, potentially sites of chymotrypsin, are highlighted in black through hFXN90–210 amino acid sequence. hFXN variants were incubated at 25°C with chymotrypsin at mass ratios of 1∶200 (protein: protease), in buffer 20 mM Tris·HCl, 100 mM NaCl, 1 mM EDTA, pH 7.0. The reaction was stopped by addition of 0.2% TFA and 1 mM PMSF. Samples were kept at −70°C until analysis by SDS–PAGE and RP–HPLC, followed by MALDI or by ESI–MS. A secondary structure scheme for residues 90–210 taken from PDB ID: 1EKG is shown on top.
(TIF)

Figure S7 The application of COREX/BEST (green) and FOLDX (blue) to calculate the the unfolding probability per hFXN residue and the contribution per residue to the protein stability, respectively. In both cases the hFXN structure input used was PDBID = 1EKG. More importantly, the algorithm COREX/BEST identified the loop 1 as the section of hFXN with the highest probability of experiencing local unfolding. FOLDX showed L198, L200 and L203 of the CTR; and residues L182, L186 and L190 of the C-terminal α-helix established stabilizing interactions.
(TIF)

Figure S8 Frustratograph on hFXN: local frustration calculated for pdb code 1EKG [40]. The protein backbone is displayed as gray ribbons for residues 90–195 and blue for residues 96–210. The direct inter-residue interactions with solid lines and the water-mediated interactions with dashed lines. Minimally frustrated interactions are shown in green, highly frustrated contacts in red, neutral contacts are not drawn.
(TIF)

Figure S9 Folding transition for the CTR. (A) Energy profile for the CTR and the hFXN90–210 protein as a function of the native contacts formed, calculated at the melting temperature of the protein. (B) Energy profile for the CTR and hFXN90–210 as a function of the native contacts formed, calculated at the melting temperature of the CTR. (C) Calorific capacity at constant volume for the CTR and the hFXN90–210. The peak represents the melting temperature for the bound/unbound reaction of the CTR with the rest of the protein. The inset shows a broader look at both transitions. Black lines represent the reactions for the whole protein while green lines represent the reactions for the CTR.
(TIF)

Figure S10 Contact matrix of the native and transition state ensembles sampled through the structure based model simulations. Each dot represents a native contact between the amino acid residue in x-axis and the amino acid residue in y-axis. The color range in the right represents the probability of formation of each contact. (A) Contact matrix for the native state ensemble. (B) Contact matrix for the transition state ensemble.
(TIF)

Figure S11 Unfolding kinetics of hFXN90–210 and hFXN90–195 followed by tryptophan fluorescence emission intensity. The unfolding reactions were performed at 20°C in buffer 20 mM Tris-HCl, 100 mM NaCl, 1 mM EDTA, pH 7.0. GdmCl was added as the chaotropic agent. The concentrations of GdmCl in the experiment were set to establish conditions where the difference in free energy between native and unfolded states is the same for both variants. If native and unfolded states of each variant are located at the same free energy level (inset), then the difference in the unfolding speed would be related to a difference in the transition barrier (?G_NTS_). The hFXN90–195 and hFXN90–210 unfolding reactions are shown in red and black dashed lines, respectively. The reactions were started by manual dilution of protein from 0 to 1.4, in the former, and 2.5 M GdmCl, in the latter case. In red and black solid lines the native signals, dilutions of proteins in the absence of denaturant agent were made for these experiments. Excitation and emission were at 295 and 346 nm, respectively.
(TIF)

Figure S12 Iron induced aggregation of hFXN90–195 (red line) and hFXN90–210 (black line) followed by light scattering. The assay was performed at 20°C in buffer 50 mM HEPES, pH 7.0. Iron (Fe^3+^) and protein concentration were 50 and 250 µM, respectively. At the 5 min mark, FeCl_3_ solution was added from a 25 mM stock solution prepared in 0.1 N HCl (arrow) and the change in OD at 350 nm was recorded. In blue, the iron was added to a 250 µM EDTA solution. In blue, the iron was added in the absence of protein to the solution buffer 50 mM HEPES, pH 7.0
(TIF)

Table S1 Concentration dependence analysis of sedimentation, at 42000 rpm and 20°C, of hFXN 90–195 and hFXN90–210, in 10 mM Tris-HCl, 100 mM NaCl, pH 7.0. The SV profiles of both variants at the three concentrations were analyzed in terms of one non-interacting species. The species is characterized by the absorbance signal at 280 nm (which is proportional to concentration c), a sedimentation coefficient s, and an apparent diffusion coefficient D_app_. The absorbance signal and s obtained by the non interacting species analysis are (within experimental error) those of the c(s) analysis. Linear regressions of s^−1^(c) and D_app_(c) provides values for sedimentation and diffusion at infinite dilution, s_0_ and D_0_. Molecular mass M and R_S_ are obtained from s_0_ and D_0_ through Svedberg and Stokes-Einstein equations respectively.
(DOC)

Table S2 Native contacts between the CTR and the rest of the protein as studied by structure-based model simulations.
(DOC)
