# Supplementary figures and images for: Protein Stability and Dynamics Modulation: The Case of Human Frataxin
Source: PLoS One. 2012 Sep 25;7(9):e45743. doi: 10.1371/journal.pone.0045743 (PMC3458073; doi:10.1371/journal.pone.0045743)

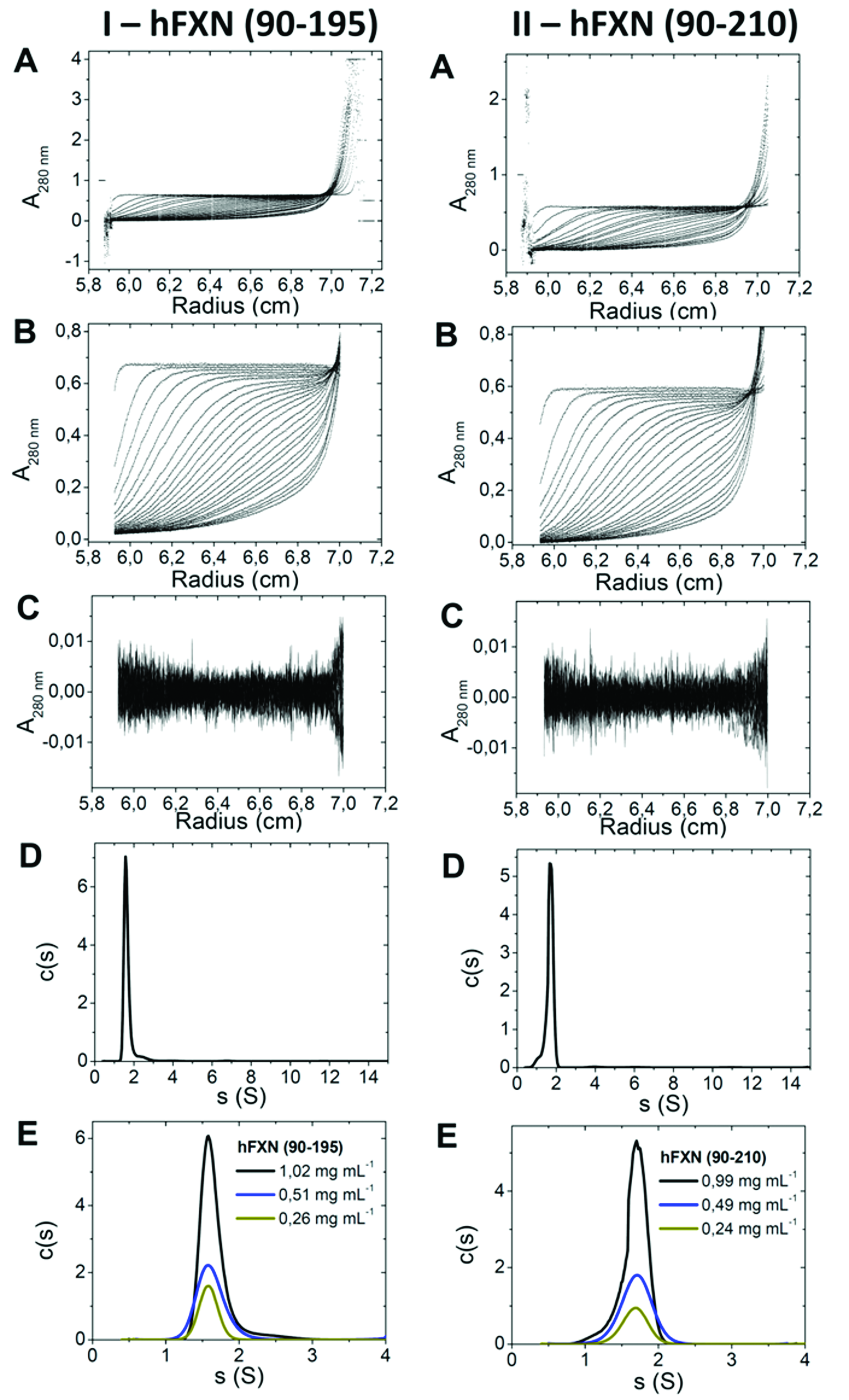

Supplement: Figure S1 — Analytical ultracentrifugation. Sedimentation velocity of hFXN90–195 (left panels) and hFXN90–210 (right panels) at 42000 rpm and 20°C, in 10 mM Tris-HCl, 100 mM NaCl, pH 7.0. (A) Selection of raw data for both proteins at 1 mg/mL. (B) Superposition of experimental (dots) and fitted (continuous line) profiles corrected for all systematic noise for both variants at 1 mg/mL. The last profiles correspond to 16 h of sedimentation. The fit was obtained from the c(s) analysis of the SEDFIT program. For both proteins, the Lamm equation was simulated for 300 particles in the ranges (0.4 S, 15 S) and (0.4 S, 4 S), with a partial specific volume mL g−1 and a frictional ratio f/fmin = 1.25 (which corresponds to a globular, usually hydrated, macromolecule). (C) Superposition of the differences between the experimental and fitted curves. (D) Corresponding c(s) distribution in the range 0.4–15 S for both variants at 1 mg/mL. The signal was normalized to 1 cm optical path length. (E) Superposition of the c(s) distributions for different concentrations of both proteins in the range 0.4–4 S, corresponding to more than 96% of the total signal. The signal was normalized to 1 cm optical path length. (TIF) [file pone.0045743.s001.tif]

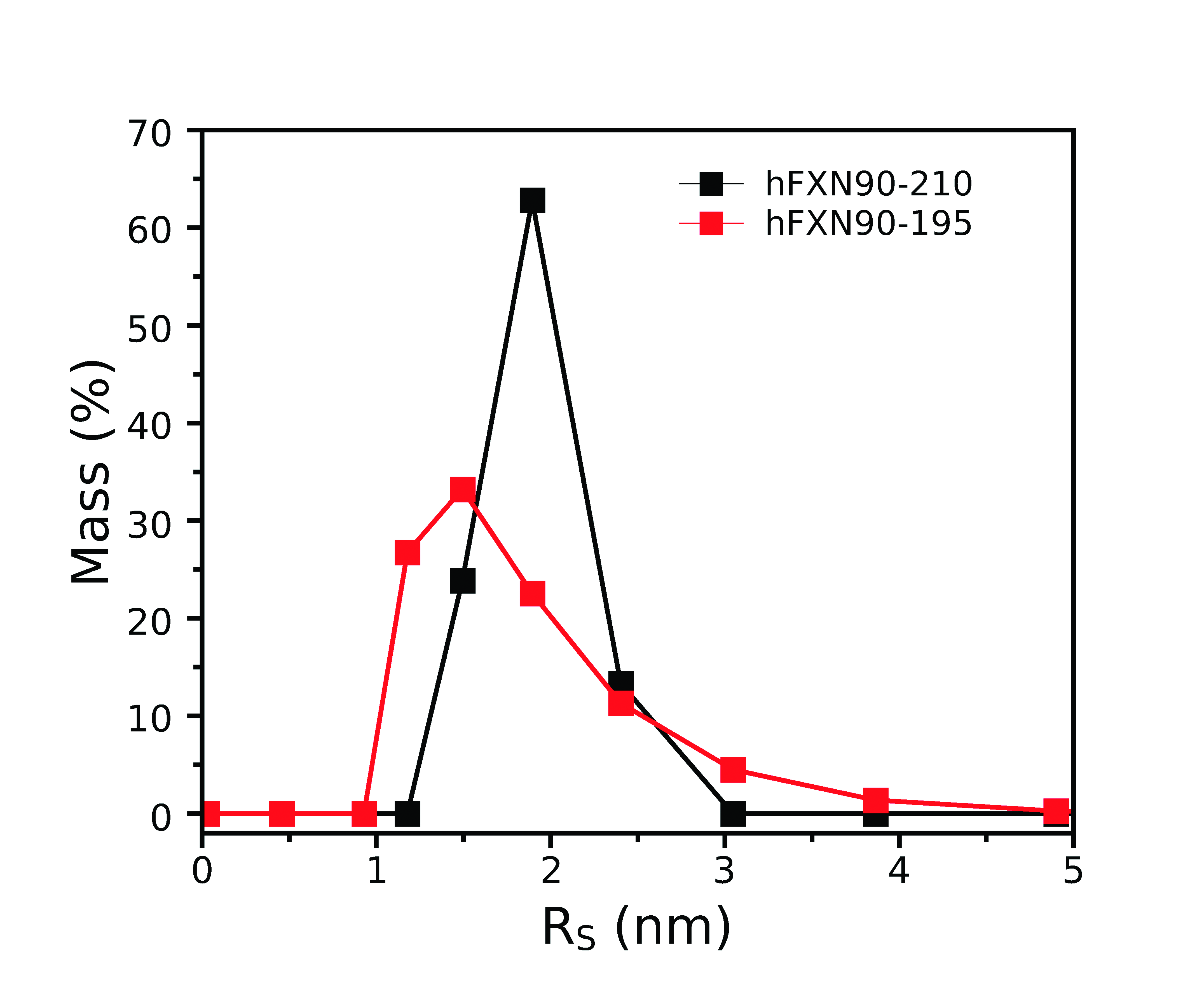

Supplement: Figure S2 — Size distribution of hFXN variants as determined by DLS. Experiments were performed in batch mode at 25°C. Samples of 50–100 µL were filtered by 0.22 µm and centrifuged for 20 min at 10000 rpm at 4.0°C. Size distribution by mass was determined using isotropic spheres as the model. Distributions for hFXN90–210 and hFXN90–195 are shown in black and red, respectively. Proteins (at 1 mg/mL in buffer 20 mM Tris-HCl, 100 mM NaCl, 1 mM EDTA, pH 7.0) were analyzed in a standalone dynamic light scattering instrument (DynaPro NanoStar from Wyatt Technology). (TIF) [file pone.0045743.s002.tif]

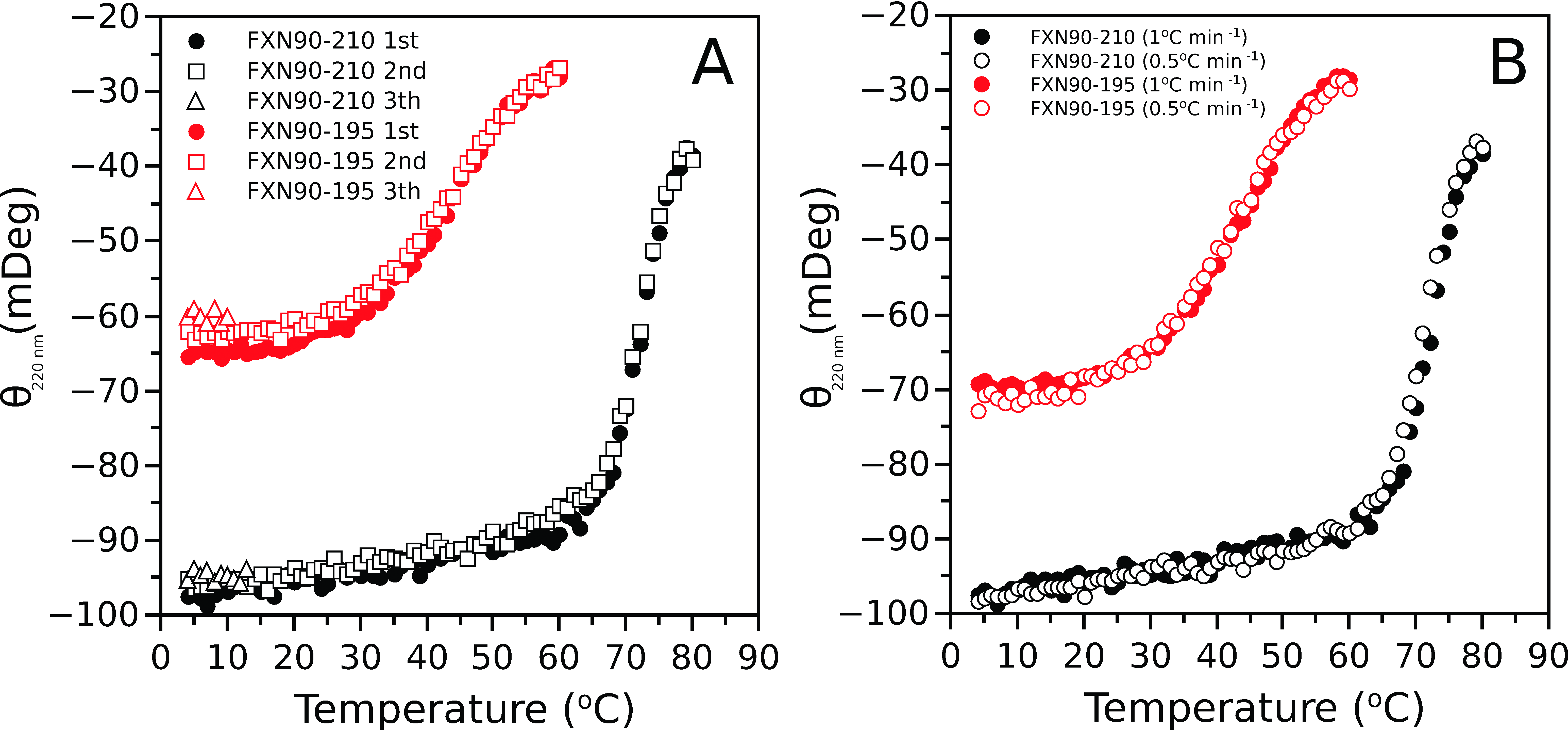

Supplement: Figure S4 — Reversibility of the temperature unfolding reactions for hFXN variants. (A) Transitions were followed by far-UV CD from 4 to 60°C and 4 to 80°C for hFXN90–195 and hFXN90–210, respectively. When proteins reach the highest temperature they were cooled to 4°C in a fast way, without control of the cooling rate. The signal recovery was 96%, and 99% for hFXN90–195 and hFXN90–210, respectively. The superposition of the consecutive unfolding curves (scan (circles), rescan (squares) and the starting points of the re-rescan (triangles)) are shown for hFXN90–195 and hFXN90–210 in red and black, respectively. The rate was 1°C/min. (B) Unfolding curves for each variant were performed at two different rates = 1.0°C min−1 and 0.5°C min−1, filled and empty symbols, respectively. (TIF) [file pone.0045743.s004.tif]

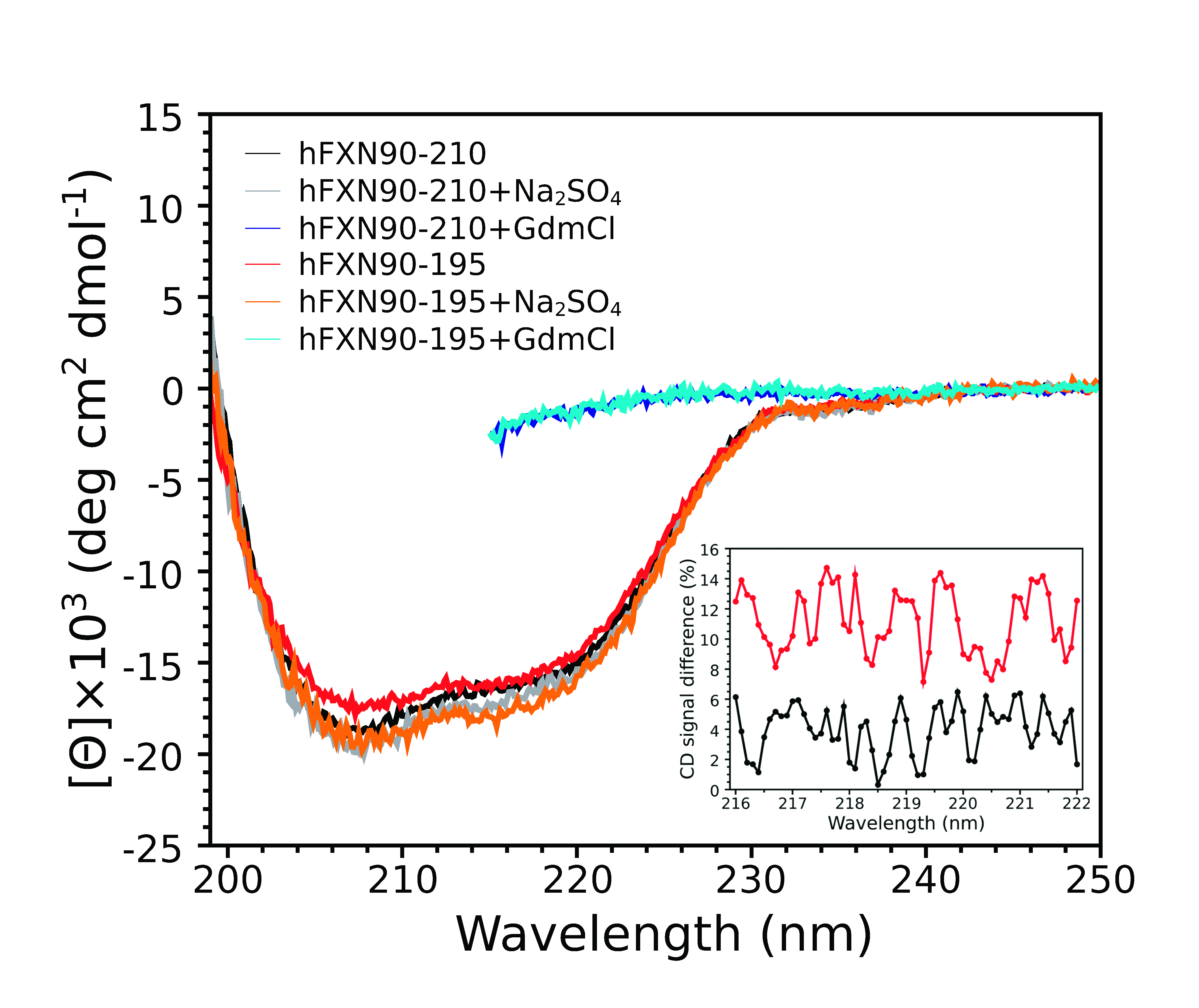

Supplement: Figure S5 — Effect of sodium sulfate on the hFXN90–195 conformation. Far-UV CD spectra of hFXN90–210 (gray) and hFXN90–195 (orange) were acquired in the presence of 200 mM Na2SO4 or in the absence of the osmolyte (FXN90–210 in black, and hFXN90–195 in red). In addition, spectra of the unfolded states of both proteins were acquired in the presence of 5.0 M GdmCl. The inset shows the CD signal difference (%) upon Na2SO4 addition. Buffer was 20 mM sodium phosphate, 100 mM NaCl, pH 7.0 and the experiment was performed at 25°C. (TIF) [file pone.0045743.s005.tif]

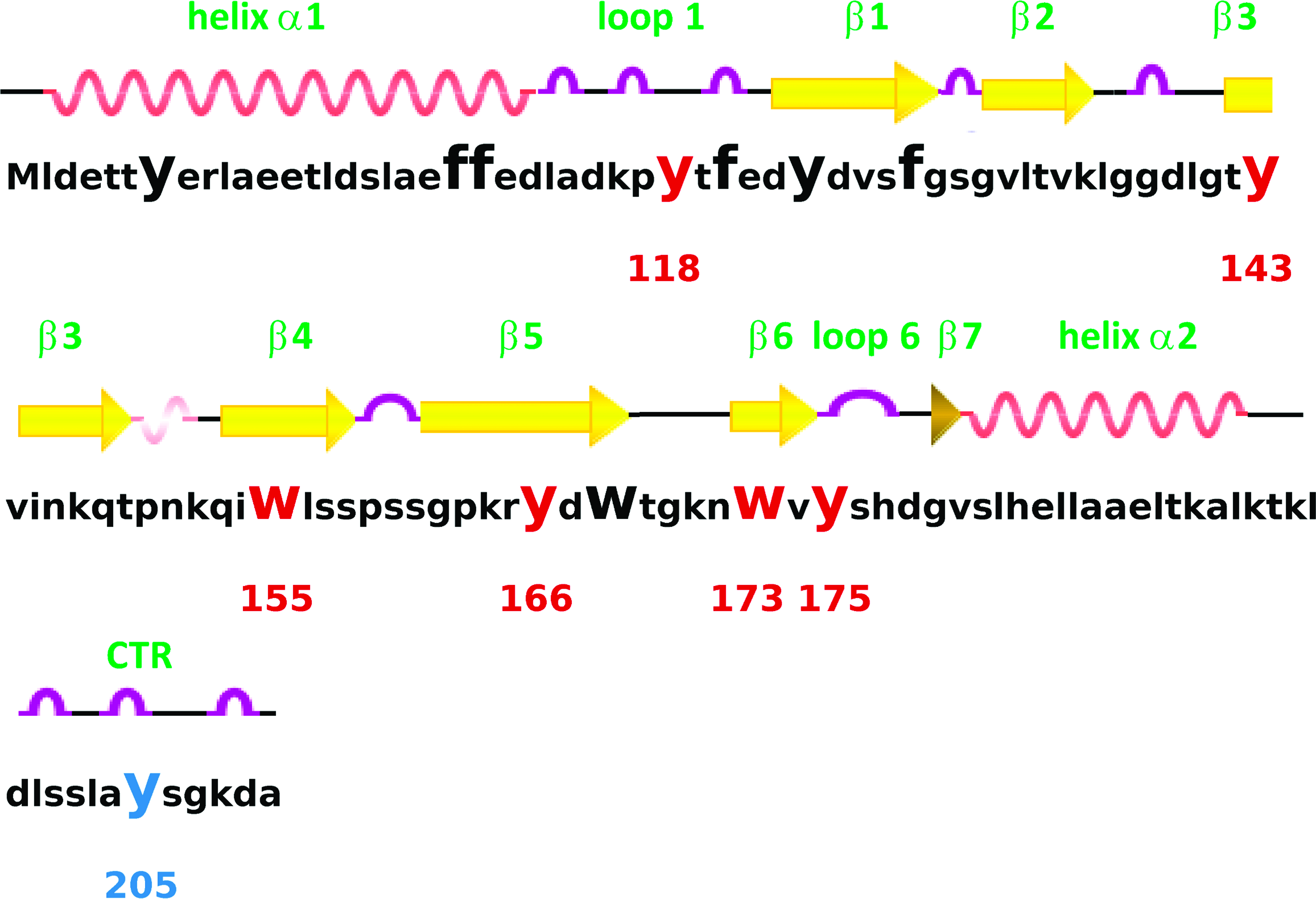

Supplement: Figure S6 — Proteolytic sites observed between 20 s and 5 min in hFXN90–195 (red) and hFXN90–210 (blue), respectively. The other aromatic residues, potentially sites of chymotrypsin, are highlighted in black through hFXN90–210 amino acid sequence. hFXN variants were incubated at 25°C with chymotrypsin at mass ratios of 1∶200 (protein: protease), in buffer 20 mM Tris·HCl, 100 mM NaCl, 1 mM EDTA, pH 7.0. The reaction was stopped by addition of 0.2% TFA and 1 mM PMSF. Samples were kept at −70°C until analysis by SDS–PAGE and RP–HPLC, followed by MALDI or by ESI–MS. A secondary structure scheme for residues 90–210 taken from PDB ID: 1EKG is shown on top. (TIF) [file pone.0045743.s006.tif]

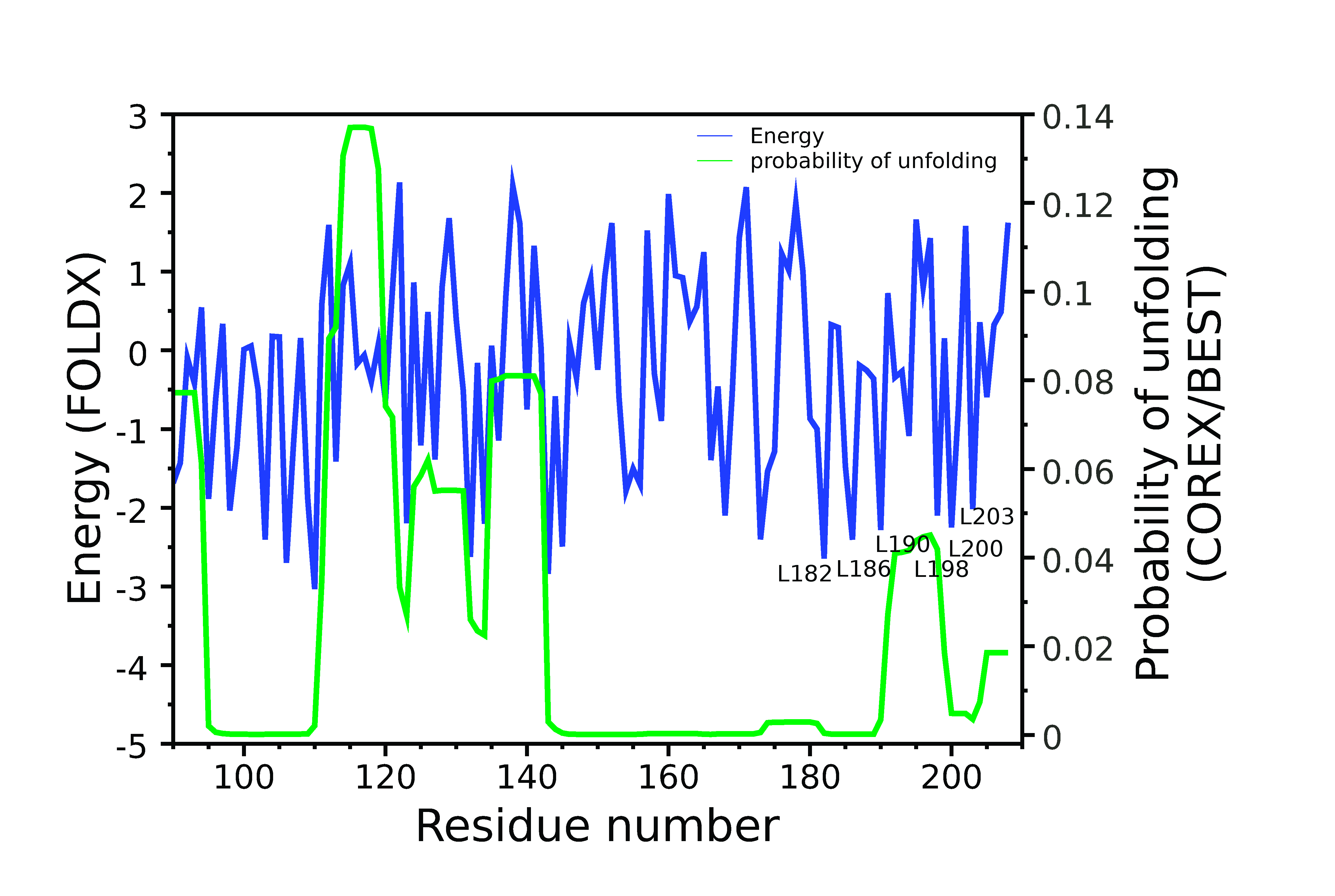

Supplement: Figure S7 — The application of COREX/BEST (green) and FOLDX (blue) to calculate the the unfolding probability per hFXN residue and the contribution per residue to the protein stability, respectively. In both cases the hFXN structure input used was PDBID = 1EKG. More importantly, the algorithm COREX/BEST identified the loop 1 as the section of hFXN with the highest probability of experiencing local unfolding. FOLDX showed L198, L200 and L203 of the CTR; and residues L182, L186 and L190 of the C-terminal α-helix established stabilizing interactions. (TIF) [file pone.0045743.s007.tif]

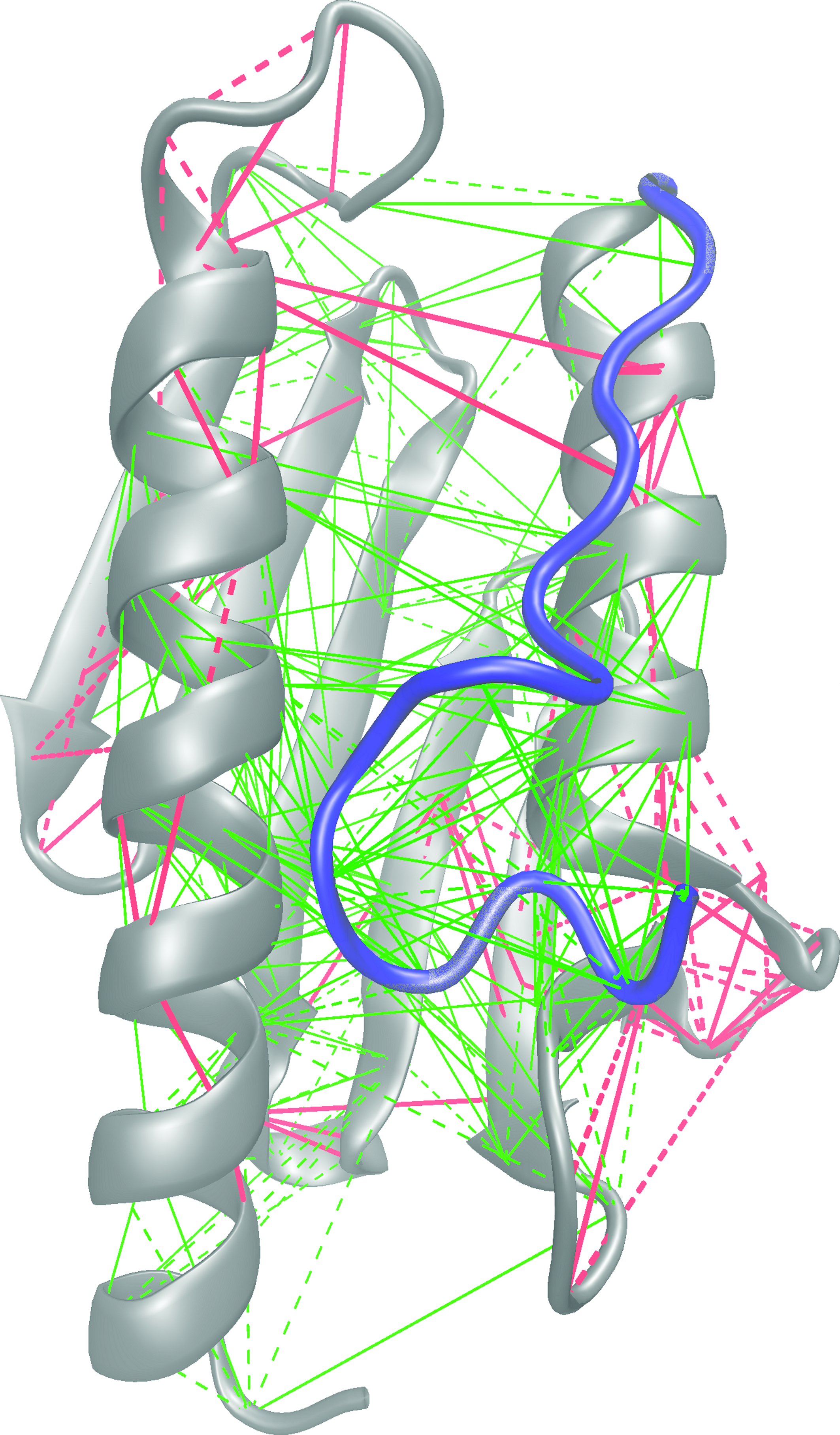

Supplement: Figure S8 — Frustratograph on hFXN: local frustration calculated for pdb code 1EKG [40]. The protein backbone is displayed as gray ribbons for residues 90–195 and blue for residues 96–210. The direct inter-residue interactions with solid lines and the water-mediated interactions with dashed lines. Minimally frustrated interactions are shown in green, highly frustrated contacts in red, neutral contacts are not drawn. (TIF) [file pone.0045743.s008.tif]

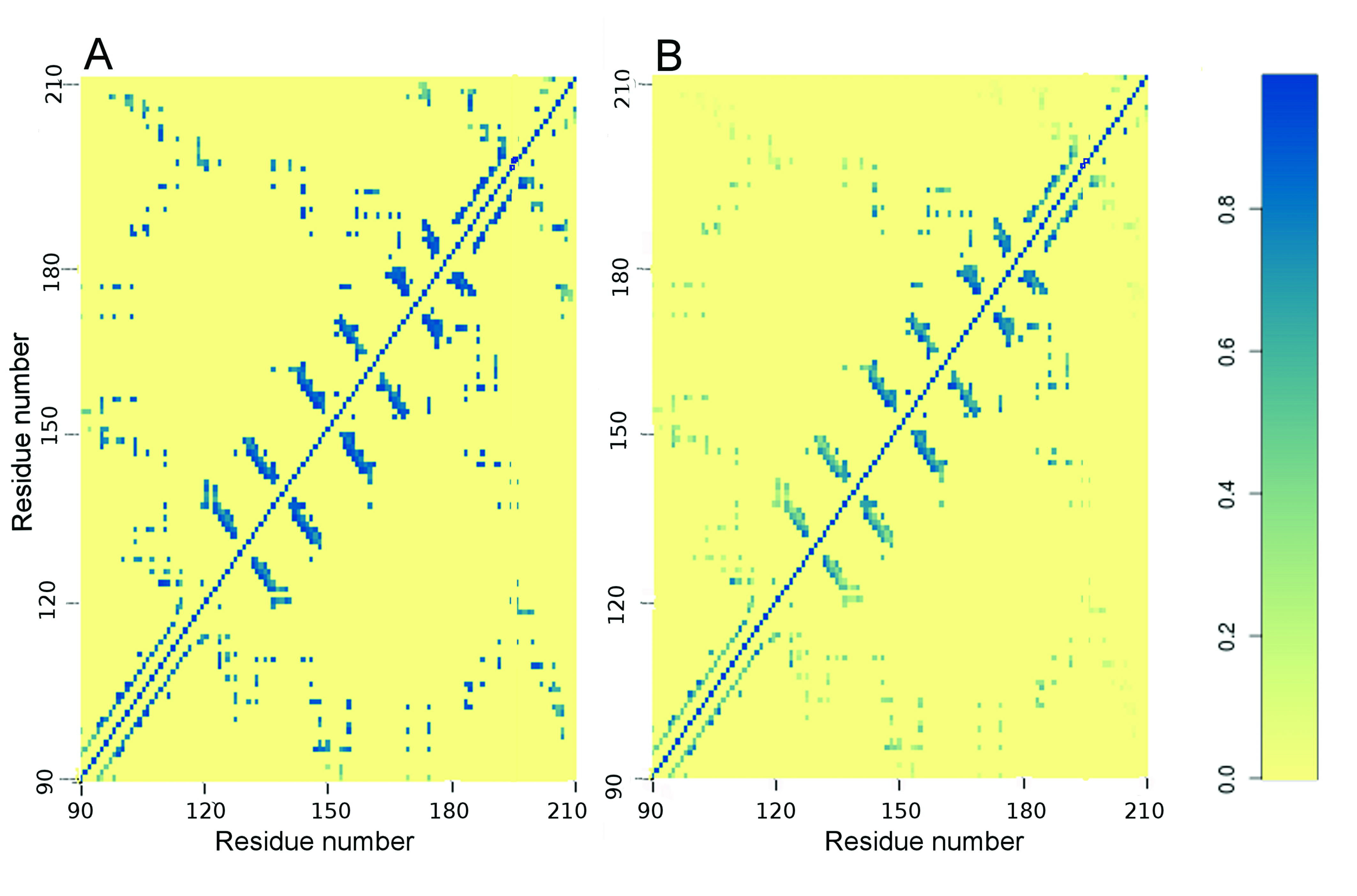

Supplement: Figure S10 — Contact matrix of the native and transition state ensembles sampled through the structure based model simulations. Each dot represents a native contact between the amino acid residue in x-axis and the amino acid residue in y-axis. The color range in the right represents the probability of formation of each contact. (A) Contact matrix for the native state ensemble. (B) Contact matrix for the transition state ensemble. (TIF) [file pone.0045743.s010.tif]

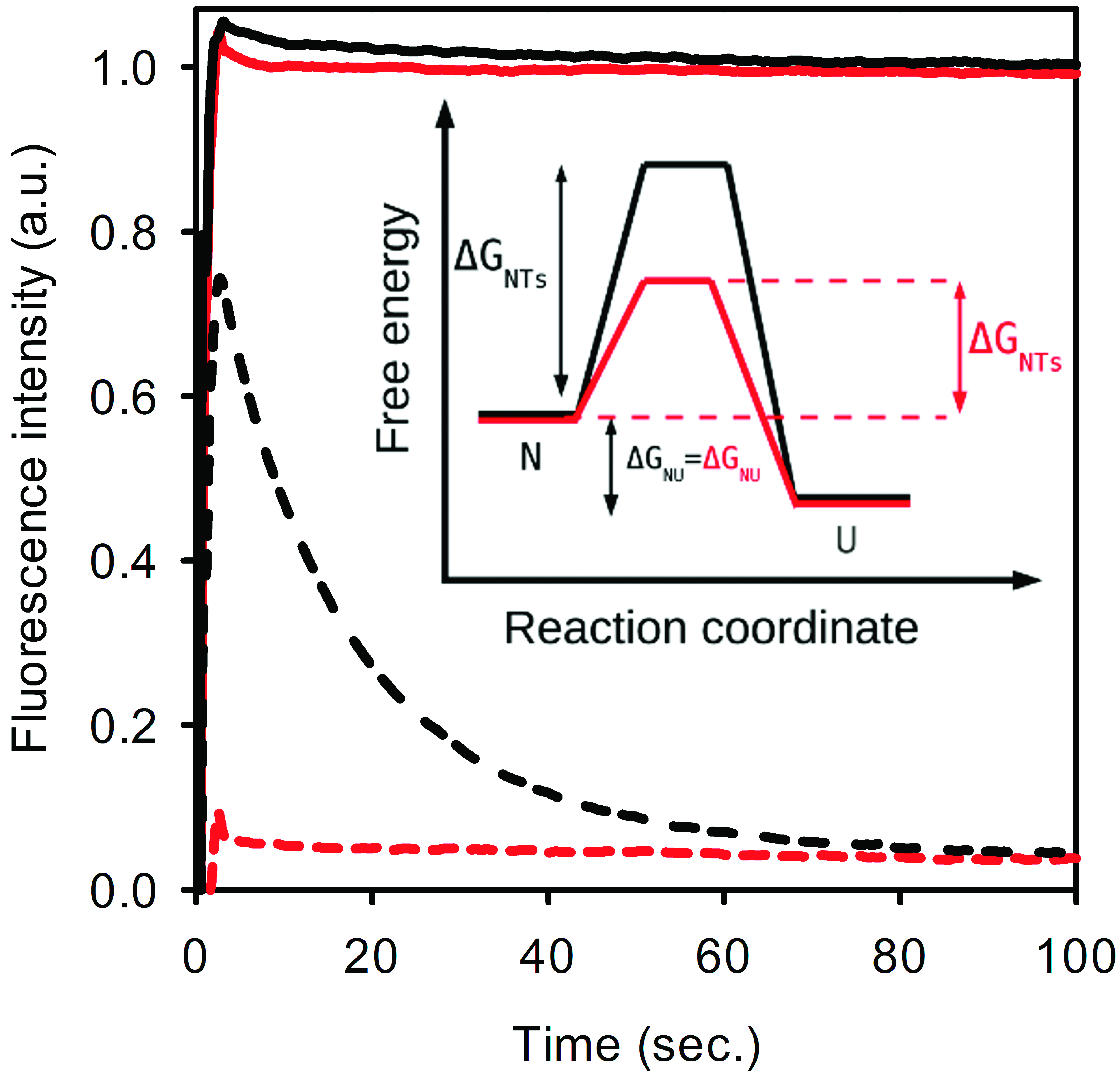

Supplement: Figure S11 — Unfolding kinetics of hFXN90–210 and hFXN90–195 followed by tryptophan fluorescence emission intensity. The unfolding reactions were performed at 20°C in buffer 20 mM Tris-HCl, 100 mM NaCl, 1 mM EDTA, pH 7.0. GdmCl was added as the chaotropic agent. The concentrations of GdmCl in the experiment were set to establish conditions where the difference in free energy between native and unfolded states is the same for both variants. If native and unfolded states of each variant are located at the same free energy level (inset), then the difference in the unfolding speed would be related to a difference in the transition barrier (?GNTS). The hFXN90–195 and hFXN90–210 unfolding reactions are shown in red and black dashed lines, respectively. The reactions were started by manual dilution of protein from 0 to 1.4, in the former, and 2.5 M GdmCl, in the latter case. In red and black solid lines the native signals, dilutions of proteins in the absence of denaturant agent were made for these experiments. Excitation and emission were at 295 and 346 nm, respectively. (TIF) [file pone.0045743.s011.tif]

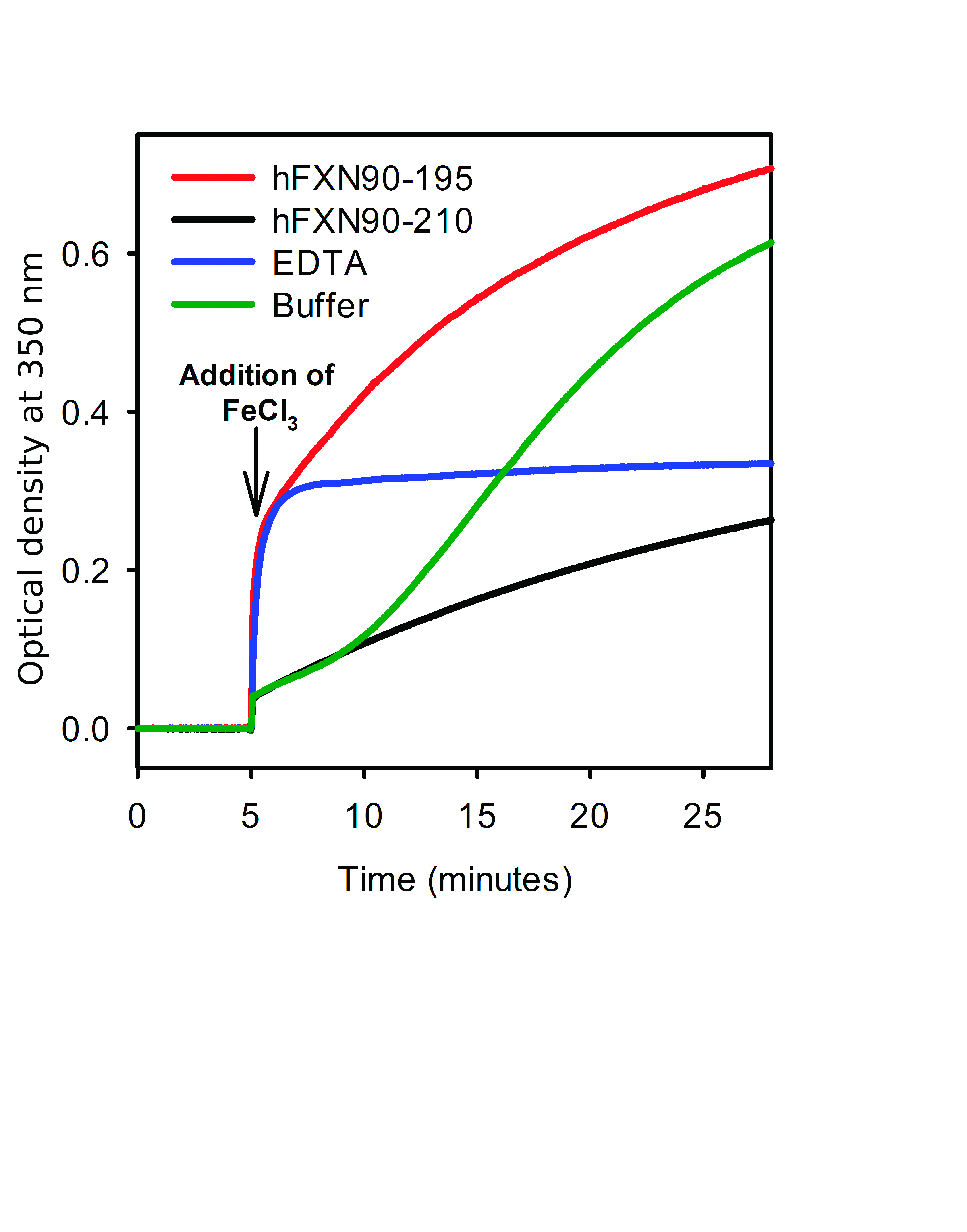

Supplement: Figure S12 — Iron induced aggregation of hFXN90–195 (red line) and hFXN90–210 (black line) followed by light scattering. The assay was performed at 20°C in buffer 50 mM HEPES, pH 7.0. Iron (Fe3+) and protein concentration were 50 and 250 µM, respectively. At the 5 min mark, FeCl3 solution was added from a 25 mM stock solution prepared in 0.1 N HCl (arrow) and the change in OD at 350 nm was recorded. In blue, the iron was added to a 250 µM EDTA solution. In blue, the iron was added in the absence of protein to the solution buffer 50 mM HEPES, pH 7.0 (TIF) [file pone.0045743.s012.tif]
