# Supplementary material for: Protein Stability and Dynamics Modulation: The Case of Human Frataxin
Source: PLoS One. 2012 Sep 25;7(9):e45743. doi: 10.1371/journal.pone.0045743 (PMC3458073; doi:10.1371/journal.pone.0045743)
Supplement: Table S1 — Concentration dependence analysis of sedimentation, at 42000 rpm and 20°C, of hFXN 90–195 and hFXN90–210, in 10 mM Tris-HCl, 100 mM NaCl, pH 7.0. The SV profiles of both variants at the three concentrations were analyzed in terms of one non-interacting species. The species is characterized by the absorbance signal at 280 nm (which is proportional to concentration c), a sedimentation coefficient s, and an apparent diffusion coefficient Dapp. The absorbance signal and s obtained by the non interacting species analysis are (within experimental error) those of the c(s) analysis. Linear regressions of s−1(c) and Dapp(c) provides values for sedimentation and diffusion at infinite dilution, s0 and D0. Molecular mass M and RS are obtained from s0 and D0 through Svedberg and Stokes-Einstein equations respectively. (DOC) [file pone.0045743.s013.doc]

**Table S1.** Concentration dependence analysis of sedimentation, at 42000 rpm and 20ºC, of hFXN 90-195 and hFXN90-210, in 10 mM Tris-HCl, 100mM NaCl, pH 7.0. The SV profiles of both variants at the three concentrations were analyzed in terms of one non-interacting species. The species is characterized by the absorbance signal at 280 nm (which is proportional to concentration c), a sedimentation coefficient s, and an apparent diffusion coefficient D_app_. The absorbance signal and s obtained by the non interacting species analysis are (within experimental error) those of the c(s) analysis. Linear regressions of s^‑1^(c) and D_app_(c) provides values for sedimentation and diffusion at infinite dilution, s_0_ and D_0_. Molecular mass M and R_S_ are obtained from s_0_ and D_0_ through Svedberg and Stokes-Einstein equations respectively.

| **Variant** | **S_0_**  **(S)** | **D_0_**  **(10^-7^cm^2^ s^-1^)** | **M**  **(kDa)** | **R_S_**  **(nm)** |
| --- | --- | --- | --- | --- |
| **hFXN90-195** | 1.55 ± 0.01 | 11.9 ± 0.1 | 12.0 ± 0.2 | 1.8 ± 0.1 |
| **hFXN90-210** | 1.70 ± 0.01 | 11.4 ± 0.1 | 13.7 ± 0.2 | 1.9 ± 0.1 |

Errors are estimated from the linear regression analysis of data and propagated according to the rules of errors.
