# Supplementary material for: Protein Stability and Dynamics Modulation: The Case of Human Frataxin
Source: PLoS One. 2012 Sep 25;7(9):e45743. doi: 10.1371/journal.pone.0045743 (PMC3458073; doi:10.1371/journal.pone.0045743)
Supplement: Table S2 — Native contacts between the CTR and the rest of the protein as studied by structure-based model simulations. (DOC) [file pone.0045743.s014.doc]

**Table S2.** Native contacts between the CTR and the rest of the protein as studied by structure-based model simulations.

| **Region in the hFXN** | **Contact formed**  **between residues** |
| --- | --- |
| **α1** | 97-205 |
|  | 98-205 |
|  | 101-204 |
|  | 101-205 |
|  | 102-205 |
|  | 102-204 |
|  | 102-203 |
|  | 105-203 |
|  | 105-204 |
|  | 105-202 |
|  | 106-203 |
|  | 106-200 |
|  | 109-203 |
|  | 109-199 |
|  | 109-200 |
|  | 109-198 |
|  | 113-198 |
| **α2** | 183 -206 |
|  | 183-200 |
|  | 183-203 |
|  | 183-205 |
|  | 183-204 |
|  | 184-206 |
|  | 186-198 |
|  | 186-200 |
|  | 187-198 |
|  | 187-200 |
|  | 187-199 |
|  | 190-198 |
|  | 190-195 |
|  | 190-196 |
|  | 190-200 |
|  | 191-198 |
|  | 191-197 |
|  | 191-196 |
|  | 191-195 |
|  | 194-198 |

| **Region in the hFXN** | **Contact**  **between residues** |
| --- | --- |
|  | 118-196 |
| **Loop1** | 118-195 |
|  | 118-198 |
|  | 118-197 |
|  | 168-205 |
|  | 171-205 |
| **Loop6/** | 171-207 |
| **β6/ Loop7** | 172-205 |
|  | 172-206 |
|  | 172-207 |
|  | 173-205 |
|  | 173-206 |
|  | 173-204 |
|  | 181-206 |
| **CTR** | 199-203 |
